# Functional Feeds Reduce Heart Inflammation and Pathology in Atlantic Salmon (*Salmo salar* L.) following Experimental Challenge with Atlantic Salmon Reovirus (ASRV)

**DOI:** 10.1371/journal.pone.0040266

**Published:** 2012-11-30

**Authors:** Laura Martinez-Rubio, Sofia Morais, Øystein Evensen, Simon Wadsworth, Kari Ruohonen, Jose L. G. Vecino, J. Gordon Bell, Douglas R. Tocher

**Affiliations:** 1 Institute of Aquaculture, School of Natural Sciences, University of Stirling, Stirling, Scotland, United Kingdom; 2 Norwegian School of Veterinary Science, Oslo, Norway; 3 EWOS Innovation AS, Dirdal, Norway; University of Maryland School of Medicine, United States of America

## Abstract

Heart and Skeletal Muscle Inflammation (HSMI), recently associated with a novel Atlantic salmon reovirus (ASRV), is currently one of the most prevalent inflammatory diseases in commercial Atlantic salmon farms in Norway. Mortality varies from low to 20%, but morbidity can be very high, reducing growth performance and causing considerable financial impact. Clinical symptoms, including myocarditis, myocardial and red skeletal muscle necrosis, correlate with the intensity of the inflammatory response. In the present study, the effects of two functional feeds (FF1 and FF2) were compared to a standard commercial reference feed (ST) in Atlantic salmon subjected to an ASRV challenge. The functional feeds had reduced levels of total lipid and digestible energy, and different levels and proportions of long-chain polyunsaturated fatty acids (LC-PUFA). The objective was to determine whether these feeds could provide effective protection by decreasing the inflammatory response associated with HSMI. Histopathology, viral load, fatty acid composition and gene expression of heart tissue were assessed over a period of 16 weeks post-infection with ASRV. The viral load and histopathology scores in heart tissue in response to ASRV infection were reduced in fish fed both functional feeds, with FF1 showing the greatest effect. Microarray hierarchical cluster analysis showed that the functional feeds greatly affected expression of inflammation/immune related genes over the course of the ASRV infection. Viral load correlated with up-regulation of pro-inflammatory genes at the early-mid stages of infection in fish fed the ST diet. Expression of inflammatory genes 16-weeks after ASRV challenge reflected the difference in efficacy between the functional feeds, with fish fed FF1 showing lower expression. Thus, severity of the lesions in heart tissue correlated with the intensity of the innate immune response and was associated with tissue fatty acid compositions. The present study demonstrated that dietary modulation through clinical nutrition had major influences on the development and severity of the response to ASRV infection in salmon. Thus, HSMI was reduced in fish fed the functional feeds, particularly FF1. The modulation of gene expression between fish fed the different feeds provided further insight into the molecular mechanisms and progression of the inflammatory and immune responses to ASRV infection in salmon.

## Introduction

Heart and Skeletal Muscle Inflammation (HSMI) was first diagnosed in 1999 [1] and has been associated with a novel piscine reovirus [2] recently defined as Atlantic salmon reovirus (ASRV) [3]. It is currently one of the most prevalent inflammatory diseases in commercial Atlantic salmon farms in Norway, with over 600 sites affected since 2003. Isolated cases have also been detected in the United Kingdom [4]. Mortality associated with HSMI varies between outbreaks; from insignificant to 20% of stock [5], but morbidity can be very high, significantly reducing growth performance, increasing feed conversion ratio, and causing considerable financial impact. Clinical symptoms include aberrant swimming behaviour (lethargic fish) believed to be associated with reduced heart function and poor circulation. Histopathologically, heart and red skeletal muscle appear to be the main organs affected showing severe inflammation. Epi-, endo-, and myocarditis and myocardial necrosis, as well as necrosis of red skeletal muscle are characteristic of the disease, although liver damage is also found [5]. These clinical symptoms are believed to correlate with the intensity of the inflammatory response, so factors modulating inflammation might influence the clinical manifestation of this disease.

One strategy to potentially control outbreaks of HSMI, or reduce the severity of the disease, is through the application of clinical nutrition and functional feeds. The concept of clinical nutrition and functional foods is well known in human nutrition. Functional foods are defined as foods that contain a component (whether a nutrient or not) that could be beneficial for the state of well-being and health, or reduce the risk of a disease, beyond the basic nutritional requirement [6]. Therefore, dietary supplementation is now a common complementary therapy, not only to improve the general health status of the population, but also for the management of common inflammatory diseases such as rheumatoid arthritis [7] or coronary diseases [8]. This type of approach is also becoming increasingly popular in aquaculture as it could potentially result in great economical savings in terms of increased productivity and lower costs of disease treatment/management [9]–[11]. At the current time HSMI vaccines are still under development and efficacy of other commercially available viral vaccines may be variable [12]. Clinical nutrition offers a valuable additional tool to reduce the impact of viral diseases.

There are many additives used in aquaculture diets including probiotics, prebiotics, immunostimulants, vitamins and nucleotides, which are included in commercial feeds to increase growth and feed conversion efficiency, as well as having positive effects on the fish immune system [11]. The replacement of fishmeal with plant-derived protein sources can lead to changes in gut flora and physiology, probably due to higher contents of non-digestible fibrous components, and increased levels of anti-nutritional factors, whose negative effects can be ameliorated by the additives mentioned above [13]. Another concern in recent years has been the replacement of dietary fish oil (FO) by vegetable oils (VO). This is a consequence of generally decreasing wild fisheries worldwide that, not only supply a declining proportion of fish for direct human consumption, but also, paradoxically, could constrain the growth of aquaculture due to reduced availability of fishmeal and FO, required for the manufacture of aqua feeds [14], [15].

It is well known that dietary polyunsaturated fatty acids (PUFA) can influence the immune response. Arachidonic acid (ARA, 20:4n-6), eicosapentaenoic acid (EPA, 20:5n-3) and docosahexaenoic acid (DHA, 22:6n-3), derived from cell membrane phospholipids, are precursors of eicosanoids, resolvins and protectins, which modulate leukocyte function and thereby influence production of inflammatory cytokines and adhesion molecules [16]. The eicosanoids derived from ARA promote pro-inflammatory responses, whereas those derived from EPA and DHA either produce a reduced inflammatory response or actually resolve (terminate) the inflammatory response [17]. Dietary supplementation with n-3 long-chain (LC) PUFA, EPA and DHA, protects against cardiovascular diseases, and may also be beneficial for some common inflammatory disorders in humans [18]. Studies have demonstrated that these LC-PUFA have the same role in immunomodulation in fish, and this has been an important concern particularly in recent years with increased energy (lipid) content of commercial diets and the aforementioned replacement of dietary FO by VO, which are devoid of LC-PUFA, but rich in n-6 PUFA [19], [20]. Such dietary replacement decreases the n-3/n-6 LC-PUFA ratio in tissues, potentially promoting the synthesis of pro-inflammatory eicosanoids, and several studies have examined whether this might affect disease resistance in farmed animals [21], . In general, fish fed diets with a high level of VO inclusion may be less resistant to infection and prone to increased severity of inflammatory responses due to the reduced n-3/n-6 PUFA ratio. Influence of dietary lipid content has also been investigated. It was reported that diets with higher lipid content may be associated with increased mortality and muscle lesions in Atlantic salmon suffering from pancreas disease, a common viral infection in salmon farming [10].

Oligonucleotide microarray analysis of tissue transcriptomes has become an increasingly popular tool in fish nutritional studies but, in addition, has also provided a global overview of the cellular and molecular events activated upon viral infections, and genome-wide changes after vaccination [23] or infection [24]. Host genes and immune responses induced after infection with different viral diseases of salmon such as infectious salmon anaemia (ISA) [25], [26] and infectious hematopoietic necrosis (IHN) virus [27] have been characterized using transcriptomic analysis. Krasnov et al. [28] identified virus responsive genes (VRG) in different viral diseases including HSMI, but there are no further descriptions of the immune response specifically related to this disease, such as the expression of host genes after infection with ASRV.

The aim of the present study was to investigate the application of a clinical nutrition strategy in Atlantic salmon subjected to experimental ASRV challenge, through the use of two potential functional feed formulations in comparison to a standard commercial feed. The standard reference diet (ST diet) contained 31% lipid derived from marine (FO and fishmeal) and terrestrial (rapeseed oil) sources. The functional feeds had reduced energy levels through lower lipid (18%), and increased levels of EPA, and increased n-3/n-6 PUFA and EPA/ARA ratios, which were achieved by increased protein (fishmeal and krill meal) (FF1 diet), or krill meal plus krill oil (FF2 diet). Krill products, especially krill oil, are good sources of EPA and DHA in a phospholipid form [29]. Dietary krill oil increased incorporation of EPA and DHA into heart phospholipids in rats [30], and decreased the inflammatory process associated with chronic diseases such as rheumatoid arthritis in humans [31]. The incorporation of anti-inflammatory LC-PUFA into heart, the main organ affected by the disease, and head kidney (the main immune organ) was assessed. In addition to histological evaluation, gene expression in heart tissue was monitored by microarray analysis over a period of 16 weeks after infection with ASRV, and viral load assessed over the later collections times (>10 weeks post challenge). Therefore, this was a nutritional study specifically designed to determine whether functional feeds could ameliorate the pathological effects of the ASRV infection, and to identify pathways/mechanisms underpinning the dietary effects in order to improve feed formulations.

## Results

### 1. The viral load and histopathology scores in heart tissue in response to ASRV infection were reduced in fish fed both functional feeds, with the most significant effects observed in fish fed FF1

**Figure 1:**
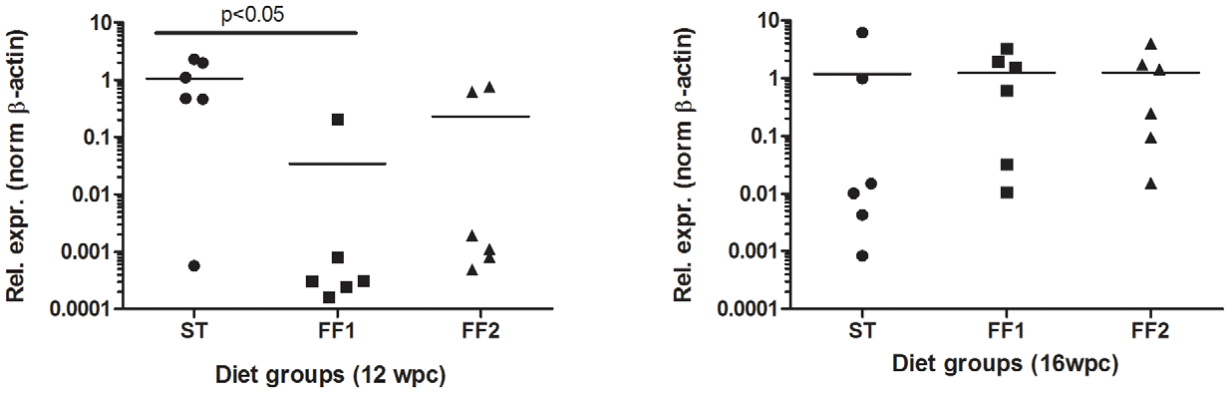
Viral load in heart tissue of the different dietary groups at 12- and 16-weeks post-challenge. Viral load was determined by quantitative real-time PCR analysis of Atlantic salmon reovirus OH-2010 strain ALV726 inner capsid protein lambda-1/VP3 gene (HM453201). Results are presented as normalised expression data (n = 6) relative to β-actin on a log scale (log_10_) with statistical analysis by Kruskal-Wallis test (non-parametric).

**Figure 2:**
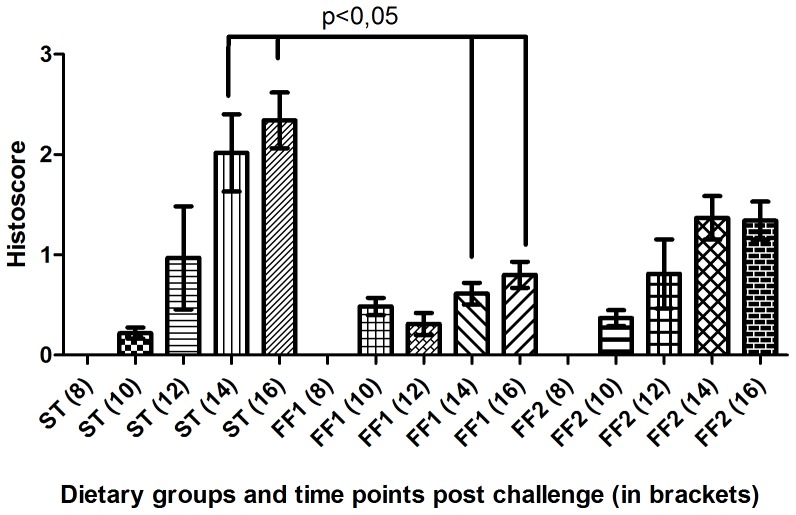
Average histoscores (± SEM) in the different dietary treatment groups at 8-, 10-, 12-, 14- and 16-weeks post-challenge. Significant differences between feed groups are indicated. N = 10 to 20 per group. Samples were scored by marking on a visual analog scale on the basis of criteria described previously by Mikalsen et al. (3) (see Table S3). A maximum score of 6 can be achieved for combined epicardial and myocardial changes. Scoring was carried out with a double-blind format.

We first used RT-qPCR to compare the viral load observed in heart tissue between the different dietary groups at later stages of the infection, when the inflammatory response typical of HSMI appeared in heart tissue in challenged fish, i.e. 12- to 16-weeks post-challenge [1], [5]. We found there was considerably higher viral load at 12-weeks post-challenge in fish consuming the ST diet compared with fish fed the two functional feeds (Fig. 1). Fish fed the ST diet showed 260-fold greater viral load (p<0.05) than fish fed the FF1 diet. Compared to the FF2 diet, relative levels were 25-fold higher in ST fish but, due to the high variability between individuals, this difference was not significant. The pathophysiological response to the infection characterised histologically by infiltration of lymphocytes and macrophages in heart tissue, reflected the pattern of viral load as had been shown earlier [3]. There was no infiltration of inflammatory or immune cells in heart tissue in any dietary group at 8-weeks post-challenge (Fig. 2). However, from 10-weeks onwards, the histopathology scores showed that the levels of cardiac inflammation were much higher at all-time points in fish consuming the ST diet compared to fish fed the functional feeds (FF1 and FF2 groups). In addition, the fish fed diet FF1 showed the significantly lowest level of cardiac inflammation when the data were combined across all time points (Fig. 2).

### 2. Fatty acid compositions of heart and head kidney tissues were associated with the severity of the inflammatory response/disease

The fact that diets modulated the viral load and inflammatory scores to the extent observed was surprising and, based on the difference in fatty acid composition between diets, we first examined the fatty acid profiles of heart and head kidney tissues (Tables 1 and 2). Generally, the fatty acid composition of these organs reflected the fatty acid composition of the diets fed to the fish (Table 3) in a similar manner to that observed in previous studies that investigated effects of diet on fatty acid profiles of heart [32] and head kidney [33]. The proportion of EPA and the EPA/ARA ratio were significantly higher in heart tissue of fish consuming the functional feeds (Table 1). In general, the overall fatty acid profile of heart tissue changed relatively little during the time-course of the infection. In contrast, we found that the fatty acid profile of head kidney tissue showed more significant differences between the three dietary groups, and the proportions of most of the LC-PUFA related to the immune response changed during the course of the infection (Table 2). For instance, the proportions of total n-3PUFA and DHA increased, and those of ARA and monoenes decreased, over the course of the infection. The proportion of EPA also decreased in fish fed the ST diet over the course of the infection but, in contrast, EPA increased in fish fed the functional feeds between weeks 8 and 16 (Table 2). Previous studies have shown that levels of EPA, ARA and DHA could determine the intensity of inflammatory response, with EPA and DHA being anti-inflammatory fatty acids and ARA pro-inflammatory [34]. In concert with this finding, our results showed that tissue levels of EPA and DHA and the EPA/ARA ratios were significantly higher in fish fed the functional feeds than in fish fed the ST diet, which was consistent with the reduced inflammatory response in heart tissue in these groups (Fig. 2).

**Table 1:** Total lipid fatty acid composition (percentage of total fatty acids) of heart from Atlantic salmon fed the different diets at 8- and 16- weeks post-infection with ASRV.

	8 weeks						16 weeks						TWO WAY ANOVA *P-value*		
*Fatty acid*	ST		FF1		FF2		ST		FF1		FF2		Diet	Week	Diet*Week
**Saturated**	25.48	±4.17	27.22	±1.92	28.45	±1.45	22.33	±0.90^a^	25.16	±1.05^b^	27.07	±1.01^c^	0.001	0.008	ns
**Mono**	26.48	±8.25	21.69	±4.19	26.74	±9.68	35.32	±7.35	26.58	±7.44	24.97	±6.90	ns	ns	ns
**20:3n-6**	0.32	±0.07^ab^	0.38	±0.06^a^	0.27	±0.06^b^	0.28	±0.02^a^	0.35	±0.05^b^	0.30	±0.03^ab^	0.005	ns	ns
**20:4n-6**	1.87	±0.76	1.95	±0.44	1.40	±0.56	1.10	±0.37	1.45	±0.42	1.53	±0.39	ns	ns	ns
**N-6 PUFA**	8.36	±1.11^a^	6.82	±0.31^b^	6.00	±0.43^b^	9.34	±0.88^a^	7.30	±0.65^b^	5.66	±0.38^c^	0.000	ns	ns
**20:5n-3**	6.21	±1.96	8.67	±1.31	6.98	±2.19	4.53	±1.27^a^	7.86	±1.39^b^	7.19	±1.21^b^	0.002	ns	ns
**22:6n-3**	27.69	±7.03	30.08	±3.40	26.67	±9.10	22.19	±6.52	27.23	±7.34	30.26	±7.29	ns	ns	ns
**N-3 PUFA**	38.64	±8.39	43.44	±4.54	38.30	±11.09	32.45	±7.20	40.28	±8.20	41.62	±8.08	ns	ns	ns
**PUFA**	48.04	±8.05	51.08	±4.22	44.81	±11.12	42.35	±6.48	48.22	±7.19	47.95	±7.70	ns	ns	ns
**EPA/ARA**	3.49	±0.65^a^	4.57	±0.77^ab^	5.21	±0.86^b^	4.19	±0.40^a^	5.62	±0.98^b^	4.83	±0.76^ab^	0.000	ns	ns
**N-3/N-6**	4.73	±1.36	6.40	±0.89	6.38	±1.83	3.56	±1.13^a^	5.62	±1.54^ab^	7.46	±1.84^b^	0.000	ns	ns

Results are means ± SD (n = 5).P-values of two-way ANOVA are presented for factors ‘diet’, ‘time’ and interaction between factors.

Values within a row at each time point with different superscript letters are significantly different as determined by one-way ANOVA.

ARA, arachidonic acid; EPA, eicosapentaenoic acid; ns, not significant (p>0.05); PUFA, polyunsaturated fatty acids.

**Table 2:** Total lipid fatty acid composition (percentage of total fatty acids) of head kidney of Atlantic salmon 8- and 16-weeks post-infection with ASRV.

	8 weeks						16 weeks						TWO WAY ANOVA *P-value*		
*Fatty acid*	ST		FF1		FF2		ST		FF1		FF2		Diet	Week	Diet*Week
**Saturated**	22.01	±0.72^a^	25.13	±1.26^b^	27.41	±0.58^c^	21.84	±0.50^a^	25.03	±0.41^b^	28.15	±0.67^c^	0.000	ns	ns
**Monounsaturated**	41.76	±2.01	40.24	±4.18	38.85	±3.44	40.44	±3.63^a^	35.15	±1.97^b^	30.38	±3.19^b^	0.000	0.000	ns
**20:3n-6**	0.24	±0.02	0.25	±0.03	0.22	±0.03	0.26	±0.03^ab^	0.30	±0.01^a^	0.24	±0.03^b^	0.002	0.002	ns
**20:4n-6**	1.31	±0.21	1.22	±0.44	1.25	±0.35	0.66	±0.14^a^	0.82	±0.08^ab^	0.88	±0.12^b^	ns	0.000	ns
**N-6 PUFA**	9.88	±0.56^a^	7.91	±0.33^b^	6.56	±0.26^c^	9.82	±0.60^a^	7.83	±0.46^b^	5.51	±0.21^c^	0.000	0.000	0.007
**20:5n-3**	4.73	±0.57	5.78	±1.33	5.81	±1.18	3.72	±0.67^a^	6.63	±0.44^b^	6.71	±0.80^b^	0.000	ns	0.027
**22:6n-3**	15.02	±1.53	14.39	±2.68	15.77	±2.26	17.47	±3.46^a^	18.26	±2.68^a^	23.69	±2.75^b^	0.014	0.000	ns
**N-3 PUFA**	25.79	±1.81	25.79	±3.93	26.52	±3.30	27.42	±3.76^a^	30.87	±2.60^ab^	35.37	±3.06^b^	0.022	0.000	ns
**PUFA**	36.23	±1.78	34.63	±4.04	33.74	±3.44	37.72	±3.15	39.82	±2.07	41.47	±2.88	ns	0.000	ns
**EPA/ARA**	3.65	±0.44^a^	4.91	±0.67^b^	4.75	±0.46^b^	5.69	±0.78^a^	8.12	±0.91^b^	7.69	±0.40^b^	0.000	0.000	ns
**N-3/N-6**	2.62	±0.26^a^	3.25	±0.41^b^	4.03	±0.38^c^	2.82	±0.52^a^	3.97	±0.56^b^	6.44	±0.76^c^	0.000	0.000	0.001

Results are means ± SD (n = 5). P-values of two-way ANOVA are presented for factors ‘diet’, ‘time’ and interaction between both factors. Values within a row with different superscript letters are significantly different as determined by one way ANOVA. ARA, arachidonic acid; EPA, eicosapentaenoic acid; ns, not significant (p>0.05); PUFA, polyunsaturated fatty acids.

**Table 3:** Total lipid fatty acid composition (percentage of total fatty acids) and lipid class composition (percentage of total lipid) of the experimental diets.

*Fatty acid*	ST	FF1	FF2
**Saturated**	23.97	36.48	31.86
**Monounsaturated**	45.73	33.10	37.05
**18:2n-6**	10.73	7.11	5.30
**20:3n-6**	0.06	0.11	0.10
**20:4n-6**	0.36	0.48	0.54
**n-6 PUFA**	11.63	8.09	6.50
**18:3n-3**	4.62	2.35	1.55
**20:5n-3**	4.85	8.76	9.53
**22:6n-3**	6.09	5.98	9.03
**n-3 PUFA**	17.83	19.83	23.32
**PUFA**	30.31	30.42	31.09
**EPA/ARA**	13.67	18.16	17.75
**N-3/N-6**	1.53	2.45	3.59
**Phospholipids**	8.94	10.77	17.30
**Triacylglycerols**	65.39	53.47	52.45
**% Lipid**	24.52	13.71	13.35

ARA, Arachidonic acid; EPA, eicosapentaenoic acid; PUFA, polyunsaturated fatty acids.

### 3. Microarray hierarchical cluster analysis showed that the functional feeds greatly affected expression of immune related genes over the course of the ASRV infection

**Figure 3:**
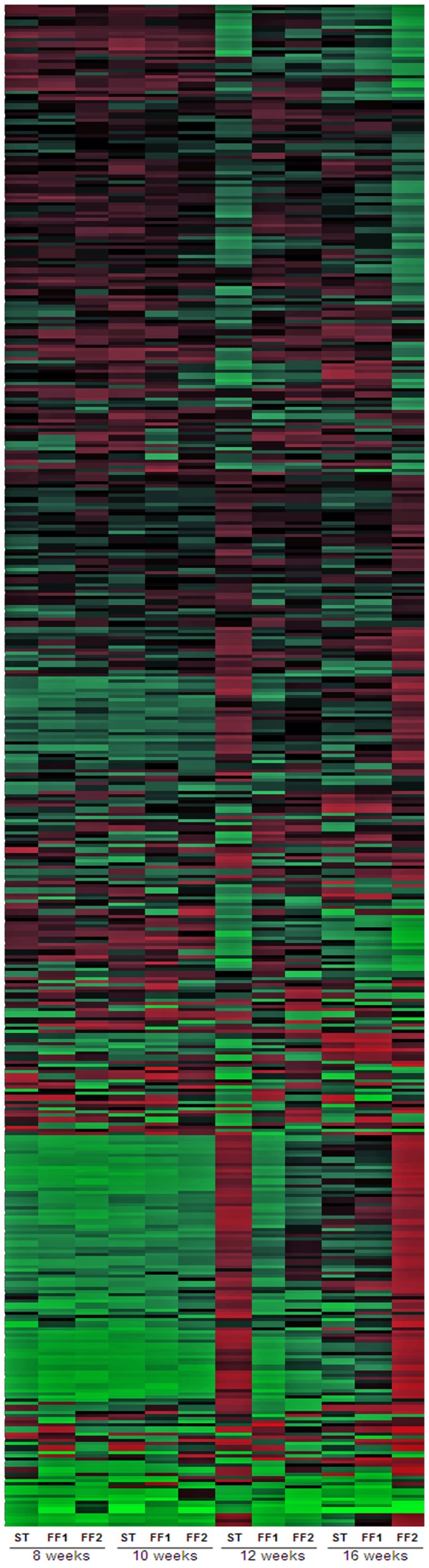
Hierarchical clustering of expression profiles for 2581 genes from the significant interaction list of the 2-way ANOVA analysis across the different dietary treatments over the time course of the infection. Individual gene expression profiles are plotted horizontally against vertical columns of each dietary group over the different time-points. Up- and down-regulation of gene expression with respect to a common reference (a pool of all the samples) are represented in red and green, respectively. Colour intensity depends on the value of the expression ratio.

We then went on to profile the transcriptomes of the heart tissue over time post challenge in an effort to fingerprint the contrasting inflammatory responses observed in the different diet groups. A hierarchical cluster analysis of heart tissue transcriptomes was performed and the results indicated clearly contrasting patterns of gene expression over the course of the infection between different dietary treatments (Fig. 3). There was a strong up-regulation (shown in red colour) of a group of genes at 12-weeks post-challenge in fish consuming the ST diet. The same group of genes was similarly strongly up-regulated at 16-weeks post-challenge in fish consuming the FF2 diet (Fig. 3, outmost lane to the right). In both cases there was concomitant down-regulation of another distinct group of genes. Based on this initial analysis, Self-Organizing Map (SOM) cluster analysis was performed to prepared two lists containing all up- and down-regulated genes from cluster groups (identified by SOM) showing these patterns of expression. The list of down-regulated features contained a set of 378 genes related to various cellular, biological and metabolic pathways including cell survival processes, regulation of apoptosis, regulation of transcription and general lipid metabolic processes (Table S1). In contrast, the list of up-regulated features contained a set of 732 genes primarily related to various aspects and stages of the immune response, including both innate and adaptive immune system responses (Table S2). To assist the interpretation of these data, the genes of the up-regulated list were sorted by fold-change comparing 8 weeks versus 16 weeks in fish fed the FF2 diet. All the genes showing an increase in expression from 8 to 12 weeks greater than or equal to 5-fold, along with a small number of other highly relevant genes with a fold-change lower than 5, were considered for more detailed discussion of the data. The 179 genes in this list (Table S2) were categorized according to the specific pathways/processes to which they are commonly associated. The effects of the dietary treatments on these pathways were analyzed to provide both an overview of the major impacts of the functional feeds and, in particular, to discriminate differences in gene expression between the two functional feeds underpinning their differential effects on HSMI. The major findings are outlined in the ensuing paragraphs.

### 4. Viral load correlated with up-regulation of pro-inflammatory genes at the early-mid stages of the infection in fish fed the ST diet

The heat map in Fig. 3 clearly indicated that timing of the immune response was affected by the functional feeds and that the ST diet at 12 weeks stands out compared to the other groups/time points. As mentioned above, viral load was highest in heart tissue of the ST diet (256× higher than FF1) at 12-weeks post-challenge (Fig. 1), which was reflected in the strong up-regulation of genes associated with viral entry and infection in ST-fed fish at that time, (shown as down-regulated in functional diets relative to ST diet in Table 4, Fig. S1A). As would be expected, the expression of genes associated with the different pathways of the innate immune response (see below) was considerably up-regulated at 12-weeks post-challenge in fish fed the ST diet (Table 5 and 6; Fig. S1B and C), particularly those involved in the interferon-inducible response (Table 6; Fig. S1C), the natural killer cells response and apoptosis (Table 7). Responses that fingerprint the cellular adaptive immune response, were in general of lower magnitude, but also markedly up regulated in the ST compared to the FF1 diet group, while lesser so compared to the FF2 diet group (Table 8). Fish in the ST diet group mount a strong, early response biased towards pro-inflammatory and early adaptive responses.

**Table 4:** Viral infection-related genes differentially expressed in heart of fish fed the functional feeds (FF1 and FF2) relative to the standard diet during the course of the infection with ASRV.

	Accession or	8 weeks		10 weeks		12 weeks		16 weeks	
BlastxHit	probe number	FF1	FF2	FF1	FF2	FF1	FF2	FF1	FF2
***Viral entry and Cellular transport***									
CD209 antigen-like protein d	Ssa#STIR16806	−1,4	−1,6	−1,2	+1,0	−3,3	−2,7	+1,3	+3,3
C-type lectin domain family 4 member e	Ssa#STIR09786	−1,4	−1,2	−1,4	+1,2	−4,1	−3,2	+1,5	+4,0
Kelch-like protein 6	DW579982	+1,2	+1,3	+1,3	+1,2	−2,6	−1,6	−1,1	+3,1
CD209 antigen-like protein A	Ssa#KSSb2272	−1,4	−1,5	+1,0	+1,3	−3,3	−2,7	+1,1	+2,7
Ecotropic viral integration site 2a	Ssa#STIR14026	−1,0	−1,1	+1,2	+1,1	−3,3	−2,1	−1,0	+2,4
Macrosialin precursor	Ssa#KSS4521	−1,3	−1,5	+1,2	+1,2	−4,8	−3,0	+1,1	+1,1
***General viral induced proteins***									
Proteasome subunit beta type-6 precursor	Ssa#STIR04422	−1,4	−1,4	+1,0	+1,1	−3,3	−2,4	+1,1	+2,3
Proteasome subunit beta type-6 precursor	Ssa#STIR02309	−1,3	−1,4	+1,0	+1,0	−3,2	−2,4	+1,2	+2,5
14 kda transmembrane protein	Ssa#STIR00068_4	−1,5	−1,4	+1,2	+1,8	−3,1	−1,6	+1,3	+3,1
Proteasome subunit beta type-6 precursor	Ssa#STIR09223	−1,4	−1,4	+1,0	+1,1	−3,2	−2,4	+1,1	+2,4
***Anti-viral protein***									
Ferritin, lower subunit putative mRNA	Ssa#STIR07882	+1,3	+1,1	+1,2	+1,5	−5,0	−2,9	−1,1	+4,1
***Retroviral DNA integration***									
Barrier-to-autointegration factor	Ssa#CL95Contig1	−1,4	−2,6	+1,6	+3,1	−12,4	−11,0	+1,9	+3,5
Prob. ATP-dependent RNA helicase DHX58	DW582483	−1,2	−2,9	+1,9	+1,6	−4,3	−5,3	+4,1	+5,5
Prob. ATP-dependent RNA helicase DHX58	DW567942	−1,5	−2,2	+1,1	+1,6	−6,3	−6,1	+1,7	+2,5

Transcripts, with probe names or accession number (when possible), are arranged by functional categories and the data are presented as the expression ratio between the functional diet group and the standard group (ST/FF1 and ST/FF2), for each time point.

**Table 5:** Innate immune system-related genes differentially expressed in heart of fish fed the functional feeds (FF1 and FF2) relative to the standard diet during the course of the infection with ASRV.

	Accession or	8 weeks		10 weeks		12 weeks		16 weeks	
BlastxHit	probe number	FF1	FF2	FF1	FF2	FF1	FF2	FF1	FF2
**Innate Immune system**									
Platelet basic protein precursor	EG823365	−2,5	−5,0	+1,1	−1,3	−17,3	−7,0	−1,2	+3,9
Platelet basic protein	EG761718	−1,9	−3,0	−1,5	−1,2	−14,7	−7,0	+1,2	+4,0
c-c motif chemokine 19 precursor	Ssa#STIR10724	−1,7	−1,5	−1,1	+1,0	−5,2	−2,7	−1,3	+6,2
Ubiquitin-like protein	BG936428	−1,2	−2,0	+1,7	+1,8	−11,1	−5,3	+1,3	+4,1
Skin mucus antibact. l-amino acid oxidase	Omy#CA377250	−4,1	−1,8	+1,1	+1,3	−27,8	−23,4	+2,1	+3,1
C-C motif chemokine 19 precursor	DW571080	−3,3	−4,0	+1,1	+1,2	−12,3	−7,2	+1,2	+3,5
Skin mucus antibact. l-amino acid oxidase	DY698830	−2,1	−2,0	+1,2	+1,4	−15,1	−9,1	+1,9	+2,7
Chemokine (c-c motif) ligand 19	Ssa#STIR04367	−2,5	−2,6	+1,3	+1,4	−5,9	−3,5	+1,5	+3,7
Chemokine (c-c motif) ligand 19	Ssa#STIR21382	−2,1	−1,9	+1,2	+1,3	−6,2	−3,4	+1,5	+3,6
Interferon regulatory factor 7	BX307182	−1,5	−1,7	+1,1	+1,2	−5,0	−3,7	+1,7	+3,3
Neutrophil cytosolic factor 2	Ssa#KSS461	−1,1	−2,8	+1,4	+2,0	−2,6	−2,0	−1,2	+1,9
Grn protein	Ssa#STIR07488	−1,3	−1,4	+1,2	+1,4	−3,7	−2,4	+1,2	+2,6
Engulfment adaptor ptb domain contain. 1	Ssa#STIR11362	+1,7	−1,9	−4,9	−5,2	−2,5	+1,3	+1,3	+4,4
Granulocyte colony-stimulating factor R prec	Ssa#STIR20316	+1,2	+1,3	−1,0	−1,6	−1,4	−1,2	−1,2	+4,7
Interferon regulatory factor 7	DY725939	−1,5	−2,0	+1,0	+1,1	−5,2	−4,0	+1,5	+2,2
Interferon regulatory factor 7	Ssa#STIR00062_4	−1,8	−2,3	+1,2	+1,4	−5,0	−4,0	+1,4	+2,2
Interferon regulatory factor 7	Ssa#TC107831	−1,6	−1,6	+1,1	+1,3	−5,9	−4,1	+1,4	+2,3
Interferon regulatory factor 7	Ssa#STIR00062_3	−1,6	−1,9	+1,1	+1,3	−5,1	−3,8	+1,4	+2,1
Bloodthirsty	Ssa#DY699301	−2,0	+1,1	−1,2	+1,0	−13,4	−5,7	+1,5	+2,4
Interferon regulatory factor 7	Ssa#STIR00062_2	−1,4	−1,7	+1,1	+1,4	−4,6	−3,8	+1,4	+2,2
Immune-related, lectin-like receptor 4	CB511660	+1,1	+1,0	+1,3	+1,2	−2,8	−1,7	−1,1	+3,0
Cysteine dioxygenase	Ssa#STIR21327	+1,1	−1,0	−1,2	+1,0	−3,1	−2,3	−1,3	+2,7
Sub-fam. B ATP-binding cassette transport 2	DW563628	−1,2	−1,2	−1,1	−1,2	−4,2	−2,9	+1,5	+1,9
Tripartite motif-containing protein 35	EG878686	−1,4	−1,3	−1,1	+1,2	−3,6	−2,8	+1,4	+2,0
Rho-related GTP-binding protein RhoG	Ssa#KSS4662	−1,2	−1,4	+1,1	+1,2	−2,9	−1,9	−1,1	+1,9
RAB26, member RAS oncogene family	Omy#BX082318	−1,9	−2,3	+1,3	+1,5	−2,7	−1,7	−1,0	+2,0
fMet-Leu-Phe receptor	Ssa#STIR17576	−1,2	−1,2	−1,1	+1,1	−2,6	−1,7	+1,1	+2,8
Bactericidal permeability-increasing protein	DW540097	−1,2	−1,6	+1,2	+1,6	−3,2	−2,2	−1,0	+2,0
TNFα-induced protein 8-like protein 2	Ssa#STIR00030_4	+1,1	−1,1	+1,1	+1,2	−2,3	−1,5	−1,1	+2,0

Transcripts, with probe names or accession number (when possible), are arranged by functional categories and the data are presented as the expression ratio between the functional diet group and the standard group (ST/FF1 and ST/FF2), for each time point.

**Table 6:** IFN I related-genes differentially expressed in heart of fish fed the functional feeds (FF1 and FF2) relative to the standard diet during the course of the infection with ASRV.

	Accession or	8 weeks		10 weeks		12 weeks		16 weeks	
BlastxHit	probe number	FF1	FF2	FF1	FF2	FF1	FF2	FF1	FF2
***JAK-STAT signal transduction pathway***									
Suppressor of cytokine signaling 1	Ssa#STIR25114	−2,1	−2,1	−1,1	+1,4	−7,0	−5,1	+1,6	+2,5
IFN-inducible protein Gig2-like	Ssa#EG876062	−1,3	−1,6	−1,1	+1,4	−5,4	−3,3	+1,9	+2,3
Suppressor of cytokine signaling 1	DW563373	−1,6	−1,4	−1,1	+1,2	−6,8	−3,8	+1,5	+2,6
N-myc (and stat) interactor	Ssa#STIR09454	−1,3	−1,5	+1,1	+1,1	−3,1	−2,3	+1,2	+2,1
IFN-inducible protein Gig2-like	Ssa#EG871163	−1,3	−1,4	−1,1	+1,1	−3,2	−2,5	+1,4	+2,1
JAK3 tyrosine kinase	Ssa#DY728848	+1,3	+1,1	+1,1	−1,2	−2,7	−1,7	−1,1	+2,0
STAT 1	Ssa#STIR06397	−1,3	−1,4	+1,0	+1,0	−3,3	−2,5	+1,4	+1,6
STAT 1α	EU016199	−1,2	−1,3	+1,1	+1,1	−3,0	−2,4	+1,5	+2,0
JAK1	Ssa#DW006200	−1,2	−1,3	+1,5	+1,3	−3,0	−2,4	+1,2	+2,0
***Interferon inducible***									
IFN inducible mx protein	U66475	−1,8	−2,3	+1,0	+1,2	−13,9	−8,9	+2,1	+3,7
IFN inducible mx protein	Ssa#STIR00154_2	−1,8	−2,2	+1,0	+1,2	−14,0	−8,3	+2,1	+3,7
Vig-2 protein	Ssa#STIR08402	−1,4	−2,5	+1,1	+1,7	−15,2	−9,2	+1,7	+2,6
Mx2 protein mRNA complete cds	Con_CANDS_07	−1,7	−1,9	+1,0	+1,1	−11,8	−7,6	+2,4	+4,0
14 kda transmembrane protein	Ssa#STIR17386	−1,7	−3,1	+4,9	+4,3	−1,2	+1,5	−1,2	+4,1
Myxovirus resistance 2	U66476	−1,5	−1,7	+1,1	+1,2	−11,3	−7,4	+2,3	+3,7
IFN inducible mx protein	Ssa#STIR00154_3	−1,6	−1,8	+1,0	+1,2	−8,3	−5,9	+1,7	+2,9
Mx1 protein mRNA complete cds	Con_CANDS_06	−1,5	−1,8	+1,0	+1,1	−8,6	−5,9	+1,7	+2,8
IFN inducible mx protein	Ssa#STIR00154_4	−1,8	−1,9	+1,0	+1,2	−8,3	−6,0	+1,8	+2,7
IFN-induced GTP-binding protein Mx	Ssa#STIR21272	−1,9	−2,5	+1,1	+1,3	−8,6	−6,9	+1,6	+2,1
vig-2 protein	Ssa#STIR10385	−1,3	−1,7	+1,1	−1,0	−7,1	−4,5	+1,4	+2,4
Mx3 protein	U66477	−1,5	−1,7	+1,0	+1,1	−7,6	−5,0	+1,8	+2,5
IFN inducible mx protein	Ssa#STIR00067_3	−1,5	−1,6	+1,0	+1,1	−7,6	−5,0	+1,8	+2,3
Cholesterol 25-hydroxylase-like protein A	DW536322	−2,1	+1,0	+2,1	+2,4	−6,5	−1,7	+1,3	+2,4
IFN-induced protein with tetratricopeptide	Ssa#STIR00073_2	−2,3	−1,8	−1,0	+1,1	−4,5	−3,9	+1,4	+1,6
IFN-induced guanylate-binding protein 1	Ssa#STIR18623	−1,0	−1,1	+1,2	+1,2	−4,6	−3,0	+1,0	+2,9
IFN-induced guanylate-binding protein 1	DY713986	−1,0	−1,4	+1,3	+1,3	−4,2	−3,6	+1,3	+2,4
14 kda transmembrane protein	Ssa#STIR22465	−1,4	−1,4	+1,2	+1,6	−2,9	−1,6	+1,2	+2,7
IFNγ induc. lysosomal thiol reductase precur	Ssa#STIR04691	−1,1	−1,3	−1,1	+1,0	−2,1	−1,5	−1,3	+2,1
IFN-induced transmembrane protein 3	CA387902	−1,4	−1,5	+1,2	+1,5	−2,4	−1,3	+1,3	+2,7
Guanylate binding protein 3	Ssa#STIR19322	−1,2	−1,2	+1,3	+1,7	−3,8	−3,3	+1,3	+2,3

Transcripts, with probe names or accession number (when possible), are arranged by functional categories and the data are presented as the expression ratio between the functional diet group and the standard group (ST/FF1 and ST/FF2), for each time point.

**Table 7:** Antiviral host responses related-genes differentially expressed in heart of fish fed the functional feeds (FF1 and FF2) relative to the standard diet during the course of the infection with ASRV.

	Accession or	8 weeks		10 weeks		12 weeks		16 weeks	
BlastxHit	probe number	FF1	FF2	FF1	FF2	FF1	FF2	FF1	FF2
***Acute phase***									
Serum amyloid A-5 protein	Ssa#KSS3451	−1,7	−2,2	+1,3	−1,3	−5,3	−3,0	+2,1	+4,3
Serum amyloid a-5 protein	Ssa#CK882427	−1,7	−2,3	+1,4	−1,3	−5,1	−2,9	+2,2	+4,0
CCAAT/enhancer-binding protein beta	Ssa#CL285Ctg1	−1,2	−1,2	+1,5	+2,0	−2,1	+1,0	+1,4	+2,4
LBP (LPS binding protein)/BPI like-2	Ssa#KSS5230	−1,2	−1,1	+1,1	+1,3	−3,3	−2,1	+1,0	+2,8
Cathelicidin	EG840650	+1,2	+1,3	−1,4	+1,1	−3,7	−2,0	+1,9	+5,4
***Natural killer cells***									
Granzyme A precursor	EG853597	+1,1	+1,7	+1,2	+1,6	−26,6	−9,1	−3,2	+5,5
Interleukin 18 form a, IL-18A	AJ556990	−1,0	+1,1	+1,2	+1,1	−1,2	+1,1	−1,3	+2,1
Immune-lectin-like receptor 3	Ssa#STIR08687	+1,0	−1,0	+1,2	+1,0	−1,8	−1,2	−1,0	+1,9
Interleukin 12b	Ssa#STIR00077_3	−1,2	−1,6	+1,3	+1,2	−1,4	−1,2	+1,3	+1,6
Perforin-1 precursor	CB515603	−1,2	−1,1	+1,1	−1,2	−1,4	−1,3	+1,3	+1,9
***Apoptosis***									
Mgc81823 transcript variant 2	Omy#TC159807	−1,0	−1,2	+1,1	+1,1	−7,8	−4,1	−1,2	+4,1
Cathepsin l precursor	Omy#CX262263	−1,3	−1,2	+1,1	+1,0	−8,2	−4,1	−1,1	+4,0
Cathepsin l	Ssa#STIR23260	+1,0	−1,0	+1,2	+1,1	−7,9	−4,5	−1,2	+3,8
E3 ubiquitin-protein ligase LINCR	DY697357	+2,2	−1,0	−2,0	+1,2	−10,9	−2,1	−1,4	+4,5
Caspase-14 precursor	EG868944	−1,3	−1,5	+1,0	+1,4	−6,2	−4,5	−1,2	+2,4
Caspase 14	Ssa#STIR02208	−1,4	+2,1	+1,4	−3,6	−2,0	+1,2	−4,5	+1,8
E3 ubiquitin-protein ligase RNF144A-A	EG829820	−1,7	−1,5	+1,1	+1,4	−6,0	−4,2	+1,0	+2,0
E3 ubiquitin-protein ligase RNF144A-A	EG841469	−1,3	−1,7	+1,2	+1,2	−5,9	−3,3	+1,0	+2,5
AN1, ubiquitin-like, homolog	Omy#CU065924	−1,2	−3,4	+1,1	−1,5	−1,9	+1,5	−1,4	−1,3
Ubiquitin carboxyl-terminal hydrolase iso L5	Ssa#S35587724_S	−1,9	−2,1	+1,0	+1,4	−1,5	−1,1	−1,8	+2,7
Ubiquitin specific protease 18	EG868088	−1,3	−2,1	−1,1	+1,1	−4,2	−3,5	+1,5	+1,9
Tripartite motif-containing protein 39	DW545632	−1,5	−1,8	+1,3	+1,4	−4,1	−4,0	+1,1	+2,1
Tyrosine-protein kinase Lyn	Omy#CX255862	−1,1	−1,5	+1,2	+1,2	−2,4	−1,8	−1,1	+2,0

Transcripts, with probe names or accession number (when possible), are arranged by functional categories and the data are presented as the expression ratio between the functional diet group and the standard group (ST/FF1 and ST/FF2), for each time point.

**Table 8:** IFN II-related genes differentially expressed in heart of fish fed the functional feeds (FF1 and FF2) relative to the standard diet during the course of the infection with ASRV.

	Accession or	8 weeks		10 weeks		12 weeks		16 weeks	
BlastxHit	probe number	FF1	FF2	FF1	FF2	FF1	FF2	FF1	FF2
**Induction proliferation and maduration of T-cells**									
T-cell receptor α chain V region 2B4 precur.	EG773958	−1,3	−5,2	−3,9	−2,0	−2,7	−1,4	−2,4	+3,1
T-cell receptor β chain	AJ517930	−1,1	−2,0	+2,1	+1,5	−4,8	−1,8	−1,2	+3,9
Differentially expressed in FDCP 6 homolog	Ssa#S48397573	−1,7	−1,8	−1,1	+1,1	−6,2	−3,2	+1,9	+2,2
Immediate early response gene 2 protein	DW562838	−1,5	−1,6	+3,6	+5,8	+1,1	+2,3	+1,2	−1,0
CD9 antigen	CB515563	−1,4	−1,2	−1,1	+1,2	−4,2	−3,1	+1,4	+2,5
T-cell receptor α chain V region 2B4 precur.	EG890703	−2,2	−1,6	−1,7	−1,4	−1,7	−1,4	+1,5	+3,2
Core-binding factor beta subunit	Ssa#TC107774	−1,5	−2,2	−1,2	+1,1	−2,9	−1,6	+1,0	+3,1
CD97 antigen	EG935955	−1,3	−1,3	+1,3	+1,2	−3,1	−2,3	−1,0	+2,4
Rho-related GTP-binding protein RhoG	Ssa#KSS4662	−1,2	−1,4	+1,1	+1,2	−2,9	−1,9	−1,1	+1,9
Ras homolog genemember g	Ssa#STIR25495	−1,3	−1,5	+1,1	+1,2	−2,4	−1,7	−1,0	+1,9
Ras homolog genemember g	Ssa#STIR05697	+1,1	−1,2	−1,6	+1,2	−2,9	−1,5	+1,3	+2,2
Hemopoietic cell kinase partial cds	AF321110	−1,0	−1,1	+1,2	+1,2	−2,6	−1,7	+1,1	+2,2
Tyrosine-protein kinase SRK2	Ssa#STIR23204	−1,8	−1,8	+1,2	+1,4	−3,9	−4,0	+1,3	+2,0
Galectin-9 putative mRNA	Ssa#STIR13309	−1,2	−1,3	−1,1	+1,1	−4,3	−2,5	+1,1	+1,9
Galectin like protein	Ssa#STIR12085	−1,2	−1,3	−1,1	+1,1	−4,3	−2,6	+1,1	+1,8
CD80-like protein	EG933501	+1,2	+1,1	+1,1	+1,2	−1,7	−1,3	+1,0	+2,3
C-C motif chemokine 28 precursor	Ssa#STIR03768	−1,5	−2,0	−1,7	+9,3	+2,1	−3,7	+1,5	+2,7
**Cellular immunity**									
Interferon gamma	AJ841811	−1,8	−2,1	+1,0	−1,2	−10,1	−4,4	−1,1	+6,4
Interferon gamma	Ssa#STIR00056_4	−1,7	−1,6	−1,4	−1,2	−11,1	−4,7	−1,0	+4,5
Interferon gamma 2	Omy#gi238231582	−1,3	−2,4	+1,2	+1,4	−5,8	−4,1	−1,0	+4,6
Interferon gamma	Ssa#STIR00056_3	+1,0	−1,1	+1,0	−2,0	−9,4	−5,7	−1,3	+3,0
Tripartite motif-containing protein 47	Ssa#DY714453	−1,1	−1,1	−1,4	−1,2	−5,4	−1,9	+2,2	+5,6
TRIM 16 protein	EG875618	−1,6	−1,7	−1,0	+1,3	−3,7	−3,3	+1,6	+2,4
Beta-2 microglobulin	AF180490	−1,3	−1,3	+1,0	+1,0	−2,6	−2,1	+1,1	+1,8
Tapasin-related protein	DW567893	−1,2	−1,1	−1,1	−1,1	−2,4	−1,9	+1,1	+1,4
CD83 antigen precursor	Ssa#KSS3110	−1,1	−1,0	+1,6	+1,6	−1,9	−1,6	−1,2	+1,4
CD80-like protein	EG933501	+1,2	+1,1	+1,1	+1,2	−1,7	−1,3	+1,0	+2,3
Tapasin	DQ451008	−1,3	−1,5	+1,1	+1,1	−2,6	−2,1	−1,0	+1,4
TAP2 protein	DW580644	−1,4	−1,6	+2,2	+2,7	−1,7	−2,3	−1,4	−1,1

Transcripts, with probe names or accession number (when possible), are arranged by functional categories and the data are presented as the expression ratio between the functional diet group and the standard group (ST/FF1 and ST/FF2), for each time point.

### 5. Contrasting inflammatory gene expression in fish fed the two functional feeds at 16-weeks after ASRV challenge

Sixteen weeks after challenge, the replication of the viral load had increased in fish consuming both functional feeds (Fig. 1). At this time they also had a similar pattern of expression of immune response genes (Tables 5,6,7,8,9), although the up-regulation was greater in fish fed the FF2 diet compared with those fed the FF1 diet (Fig. S1B–F). Genes related to both the innate (Table 5; Fig. S1B) and adaptive immune responses (Tables 8 and 9; Fig. S1E and F) were particularly highly expressed in fish fed the FF2 diet compared with fish fed the FF1 diet. The expression of the MHC class II gene was, however, higher in fish fed the FF1 diet than in fish fed the FF2 diet and, in both groups, the expression of this gene was much higher than in the fish fed the ST diet (Table 9). Leukotriene B_4_ receptor and arachidonate 5-lipoxygenase (ALOX) are directly related to the eicosanoid pathway and therefore the expression of their genes is likely related to levels of ARA and EPA. We found that both were up-regulated at 16-weeks in fish fed both functional feeds and interestingly the fold-changes comparing the expression of both genes between 8- and 16-weeks were twice as high in fed the FF2 diet compared to fish fed the FF1 diet (Table 10), which again concurs with the observed difference in the inflammatory profile between the two diets.

**Table 9:** Adaptive immune system related-genes differentially expressed in heart of fish fed the functional feeds (FF1 and FF2) relative to the standard diet during the course of the infection with ASRV.

	Accession or	8 weeks		10 weeks		12 weeks		16 weeks	
BlastxHit	probe number	FF1	FF2	FF1	FF2	FF1	FF2	FF1	FF2
***Innate to adaptive***									
MHC class II antigen beta chain	Ssa#STIR00007_3	−1,2	−4,5	−16,5	−4,2	−3,1	+1,2	+41,0	+15,0
MHC class II antigen beta chain	Ssa#STIR00007_4	+1,3	−2,4	−36,5	−7,4	−2,1	−1,2	+70,6	+27,4
IFNγ-inducible lysosomal thiolreductase prec.	Ssa#STIR09719	−1,1	−1,3	−1,0	+1,0	−2,1	−1,5	−1,3	+2,1
IFNγ-inducible lysosomal thiolreductase prec.	Ssa#STIR04691	−1,1	−1,3	−1,1	+1,0	−2,1	−1,5	−1,3	+2,1
Chemokine (c-x-c motif) ligand 13	Ssa#STIR03818	−2,7	−1,6	−1,1	+1,0	−4,8	−2,3	+3,8	+13,1
CC chemokine	Ssa#STIR23541	−1,5	−1,4	+1,5	−1,4	−5,0	−3,4	+1,6	+5,2
CD226 antigen	DW553192	−2,2	−2,2	+1,6	+1,2	−3,5	−1,4	+1,9	+6,5
TNF superfamily member 14	Ssa#S31975856_S	−5,9	−3,2	+1,4	−1,0	−5,2	−1,5	−1,2	+4,4
Chemokine cxcl-c1c	Ssa#STIR11364	−1,6	−1,6	+1,1	+1,2	−5,1	−3,0	+1,7	+3,5
High affinity IgG fc receptor i precursor	Ssa#STIR11789	−2,6	−1,1	−1,9	+1,4	−2,4	−3,0	+1,3	+3,2
High affinity IgE receptor subunit γ precur.	Ssa#STIR03935	−1,1	−1,2	+1,0	+1,2	−2,8	−2,0	−1,0	+2,4
High affinity IgG fc receptor i precursor	Ssa#STIR24788	−1,3	−1,5	+1,1	+1,2	−2,8	−1,8	+1,0	+2,2
High affinity IgE receptor subunit γ precur.	Ssa#STIR08428	−1,1	−1,3	+1,0	+1,2	−2,8	−2,0	−1,0	+2,4
High affinity IgE receptor subunit γ precur.	Ssa#STIR09946	−1,1	−1,3	+1,0	+1,2	−2,7	−2,0	+1,0	+2,4
High affinity IgE receptor subunit γ precur.	Ssa#STIR05473	−1,1	−1,2	+1,1	+1,2	−2,7	−1,9	−1,0	+2,3
Chemokine (c-x-c motif) receptor 3	Ssa#STIR10673	−1,1	−1,2	+1,5	+1,2	−2,7	−2,0	+1,1	+2,3
Chemokine (c-x-c motif) ligand 10	Ssa#STIR02663	−1,1	−1,1	+1,1	+1,1	−2,6	−2,3	+1,2	+2,2
C-X-C chemokine receptor type 3A, variant 1	AJ888878	+1,1	+1,0	+1,1	+1,2	−2,3	−1,7	−1,0	+2,4
**Humoral immunity**									
Interleukin 20alpha	Ssa#STIR13743	+1,0	−1,7	+1,3	+2,2	−10,6	−10,6	−2,2	+1,9
PREDICTED: similar to CD22 molecule	CU073328	+2,2	−1,2	+1,3	−1,8	−5,2	−1,4	−4,0	+1,3
Interleukin 20alpha	Ssa#STIR18005	+1,0	−1,2	+1,2	+1,1	−3,0	−2,0	−1,0	+2,2
Interleukin 10beta	Ssa#STIR00087_3	−1,7	−1,7	+1,1	+1,3	−4,1	−3,2	+1,5	+1,9
Leukocyte surface antigen CD53	EG836748	−1,0	−1,1	+1,2	+1,3	−2,3	−1,6	−1,1	+2,2
TNFR superfamily member 5	Ssa#CL366Ctg1	−1,1	−1,1	+1,2	+1,4	−2,1	−1,6	+1,2	+2,2
B-cell linker protein	Ssa#KSS4516	+1,2	+1,3	−1,0	+1,2	−1,9	−1,2	+1,4	+2,5

Transcripts, with probe names or accession number (when possible), are arranged by functional categories and the data are presented as the expression ratio between the functional diet group and the standard group (ST/FF1 and ST/FF2), for each time point.

**Table 10:** Lipid-related inflammatory pathway genes differentially expressed in heart of fish fed the functional feeds (FF1 and FF2) relative to the standard diet during the course of the infection with ASRV.

	Accession or	8 weeks		10 weeks		12 weeks		16 weeks	
BlastxHit	probe number	FF1	FF2	FF1	FF2	FF1	FF2	FF1	FF2
***Inflamatory pathways***									
Leukotriene B4 receptor 1 (LTB4-R 1)	DW558098	−1,0	+1,1	+1,2	+1,1	−2,6	−1,5	+1,4	+3,4
Arachidonate 5-lipoxygenase	Ssa#DW575444	+1,2	+1,2	+1,0	+1,1	−1,6	−1,2	−1,1	+1,9

Transcripts, with probe names or accession number (when possible), are arranged by functional categories and the data are presented as the expression ratio between the functional diet group and the standard group (ST/FF1 and ST/FF2), for each time point.

### 6. Severity of the lesions observed in heart tissue correlated with the intensity of the innate immune response

The microarray data showed that increased expression of genes related to the innate immune response correlated with higher histopathology scores. In general, this correlation was most obvious in fish fed the ST diet at 12 weeks post-challenge, with higher expression of genes involved in pathways of the virus-induced innate immune response. These included platelet basic protein, IRF7 and barrier to auto integration factor (Table 5), interferon-inducible Mx proteins, Vig-2, gig-2 and cholesterol 25-hydrolase (Table 6), granzyme A, serum amyloid A5, interleukins 12 and 18 and genes related to apoptosis (Table 7), and chemokines (Table 5 and 9) among others. Again, there was a lower intensity of this immune response in fish fed both the functional feeds, particularly FF1, which correlated with the lower histopathology scores in this group.

### 7. Innate and adaptive immune responses

Activation of the innate immune response in fish leads to the stimulation of different components of the adaptive immune response [35], [36]. Consistent with this, the data obtained in the present study show that differential upregulation of innate responses in the ST diet group at 12 weeks (Table 5, Fig. S1B) coincide with high expression of adaptive immune response genes at 12 weeks in the ST group (Table 9; Fig. S1F). Similarly FF1 and particularly FF2 have higher upregulation of innate and adaptive immune genes by 16 weeks (Table 5 and 9; Fig. S1B and F).

### 8. The microarray analyses data were fully validated by RT-qPCR of selected genes

Validation of the microarray data was performed by comparing the expression of 12 key genes showing both up- and down-regulation with a range of fold-changes from 1.1 to 13.9, across all three feed comparisons, and at two time points (12- and 16-weeks), providing a matrix of 72 comparisons. There was extremely good agreement between the microarray results and the RT-qPCR quantification with 93% of comparisons (67 out of 72) showing identical regulation with very similar fold differences (Table 11). This analysis indicates that the gene expression data obtained using the oligoarray were reliable and robust, which enabled interpretations to be made with some confidence, at least with regards to immune response-related genes.

**Table 11:** RT-qPCR validation of microarray results.

	12 weeks				16 weeks			
	FF1		FF2		FF1		FF2	
	Microarray	qPCR	Microarray	qPCR	Microarray	qPCR	Microarray	qPCR
**IL10**	−3.89	−3.73	−3.31	−2.87	+1.50	+1.32	+1.85	+1.13
**Casp14**	−6.19	−4.82	−4.50	−2.47	−1.15	−1.33	+2.39	+1.57
**TCRa**	−2.70	−2.34	−1.39	−1.32	−2.37	−1.58	+3.07	+1.26
**IRF1**	−2.45	−2.56	−1.90	−1.57	−1.10	−1.10	+1.82	+1.40
**MX1**	−13.88	−13.57	−8.87	−8.44	+2.08	+2.06	+3.66	+2.48
**INFII**	−9.36	−8.48	−5.74	−4.58	−1.28	−1.91	+3.01	+1.25
**GIG2**	−5.41	−5.59	−3.34	−4.41	+1.95	+2.06	+2.34	+1.69
**SAA**	−5.07	−3.95	−2.90	−2.43	+2.20	+1.74	+4.01	+2.00
**FLAP**	−1.50	−1.26	−1.23	+1.34	+1.12	+1.01	+2.04	+1.42
**BAF**	−12.38	−11.99	−11.02	−11.78	+1.88	+1.82	+3.47	+2.03
**B2M**	−2.59	−1.83	−2.11	−1.68	+1.11	−1.54	+1.79	−1.49
**IgER**	−2.23	−2.18	−1.68	−1.35	−1.04	−1.22	+2.03	+1.22

Values represent the expression ratios for the selected genes between the functional diet group and the standard group at 12- and 16-weeks post-infection with ASRV obtained by microarray analysis or RTqPCR.

## Discussion

This study demonstrated that a clinical nutrition approach through the use of functional feeds significantly reduced viral load in the heart tissue and resulted in significantly lower heart pathology over the course of the experiment. There was also a corresponding lower expression of inflammatory and immune markers in heart tissue of fish fed the functional feeds.

There are several studies evaluating the modulation of inflammatory responses to bacterial or viral infections in fish fed diets formulated with different lipid sources [37]–[40]. In general, diets containing high inclusion of VO, particularly n-6 PUFA-rich oils such as soybean [39] or sunflower [40], have lower n-3/n-6 PUFA and EPA/ARA ratios, which can lead to increased pro-inflammatory responses in fish. This is a consequence of the production of pro-inflammatory eicosanoids (e.g. LTB_4_, TXA_2_ and PGE_2_) derived from ARA released from cell membrane phospholipids of, especially, immune-related cells [32]. In the present study, a high quality feed with a commercial formulation served as a standard reference diet (ST), with protein supplied by a mixture of fishmeal and plant meals, and FO and rapeseed oil as the main lipid sources. Functional feeds were designed with reduced lipid content and relatively small changes in fatty acid composition focussed on increasing supply of EPA in the form of phospholipid. The hypothesis was that these changes in dietary formulation would increase the potential bioavailability of EPA by increasing its relative proportion in tissue phospholipids, and thus mitigate the inflammatory response to ASRV infection in fish fed the functional feeds. One analytical tool was the oligoarray, and the present trial clearly demonstrated the utility of the Atlantic salmon 44 k microarray for transcriptomic analysis and the evaluation of a large number of genes involved in many metabolic pathways. The oligoarray results provided an overview of when and how the immune response developed after an infection with ASRV and, especially, how changes in diet (feed formulation) influenced the magnitude and progression of these processes.

In addition, a further hypothesis was that the mechanism whereby increased EPA availability in fish fed the functional feeds could affect immune response would involve eicosanoid pathways. Although heart is the main organ affected in fish suffering HSMI, head kidney is an important immune-related tissue in fish producing macrophages, monocytes, B cells and other immune-related cells [19]. Thus, as macrophages are a major source of eicosanoids, the fatty acid profiles of both organs were determined to define the precise effects of the different feeds on key tissue compositions. Although the fatty acid profile of the heart tissue was more conserved during the course of the infection, the higher levels of EPA in the functional feeds was reflected in this organ, with the proportion of EPA and the EPA/ARA ratio being significantly higher in both groups of fish fed the FF diets. However, head kidney fatty acid profile showed more extensive differences in fatty acid composition, not only between fish fed the ST diet and fish fed the FF feeds, but also during the time course of the infection, including decreased ARA. These data confirm that salmon consuming the functional feeds did indeed display higher absolute and relative levels of EPA, which could increase its potential bioavailability for eicosanoid synthesis. The data also suggested that head kidney showed signs of a mobilization of key fatty acids, especially ARA, during the course of the infection.

The results from the histological examinations showed that fish fed the ST diet had relatively high scores 12-weeks after ASRV inoculation, as reported in previous studies describing the aetiology of HSMI [1], [5]. Histopathology scores from the ST-fed group showed that half of the fish sampled had major inflammation of the heart tissue at 12-weeks, and that the inflammatory changes were even greater at the end of the experiment. In contrast, fish fed the functional feeds were relatively unaffected by the disease at 12-weeks. Furthermore, at later time points, when inflammation was observed, it was less severe than in fish fed the ST diet. These data are consistent with our overall hypothesis that increasing dietary EPA and reducing dietary energy content would be beneficial in reducing the severity of the disease through the salmon experiencing a milder inflammatory response. However, the results also showed that the fish fed the FF1 diet showed the lowest inflammatory scores and, therefore, supplementation of EPA in the form of krill oil, as in the FF2 diet, was not more effective than simply supplying it as FO. Although the EPA/ARA ratio was higher in the FF1 diet than in the FF2 diet, the similarity in lipid content and fatty acid profiles of the two functional feeds, and the tissues of salmon fed the diets, suggests that another dietary factor could underlie the differences in immune response [41]. Therefore, the precise reason for the difference between the two functional feeds is not clear at present.

Transcriptomic analyses have been used recently to assess changes in the expression of immune-related genes in viral infections [25], [27], [42], but these studies have focussed on the early stages of the infection as, in some cases, the high virulence of the virus caused mortalities early in the infection. In the present study, the slow replication of ASRV and the low mortality previously recorded during HSMI outbreaks [1], [5] enabled us to evaluate the immune response over a much longer term. In most microarray studies, clustering methods are commonly used where a wide range of genes require organization [27], [43]. The graphical display of the gene categories, and their pattern of expression during the course of the infection, enabled clear discrimination of when, and in which dietary treatment, the major responses occurred. The cluster analysis showed that, at early stages of the infection, 8- to 10-weeks post-challenge, there were only relatively minor differences in gene expression between fish fed the different dietary treatments, whereas there was strong up-regulation of immune-related genes 12-weeks post-challenge in fish fed the ST diet compared to fish fed the FF1 and FF2 diets. Similar up-regulation of the same gene set was observed 16-weeks post-infection in fish fed the FF2 diet whereas fish fed the FF1 diet did not show a similar up-regulation of this gene set during the time-course of the experiment. Although the modification of expression of some immune related genes by functional feeds has been reported previously [11], clear differential effects of feeds on gene expression patterns, as displayed in the cluster analysis in the present study, are largely unprecedented and not previously reported in fish. The markedly different modulation of gene expression confirms that changes in dietary composition, including lipid content and source, and relatively small changes in fatty acid profiles, may influence the severity of the immune response.

The transcriptome analysis revealed a clear correlation between the severity of histopathological lesions and activation of the innate immune response in heart tissue. A similar correlation was described in previous studies on viral infections in salmon, specifically ISAV [25] and PMCV [44], the latter being the causal agent of cardiomyopathy syndrome (CMS). Furthermore, the transcriptome analysis uncovered important players in the immune response, aiding its characterization. Up-regulation of genes related to antiviral host responses was observed when the heart lesions were higher, and this also correlated with the higher viral load (12-weeks post-challenge for fish fed ST diet and 16-weeks post-challenge for fish fed the other diets, particularly FF2). Therefore, genes like barrier-to-autointegration factor, chemokine 19, platelet basic protein, gig-2, galectin-9 among others, and those genes related to apoptosis, were strongly up-regulated. Although up-regulation of similar suites of genes was reported in earlier studies on common salmon viral diseases [28], [25], [44], fold-changes as high as those obtained in the present study have not been reported previously. This could be related to improvements in oligomicroarray technology but, most likely, also to the longer duration of the present experimental design.

When viral load are high, interferon regulatory factors (IRF) are induced to activate the transcription of type I interferon (IFN) [45]. In the present study, the expression of IRF7 was up-regulated. Although type I IFNs were not present in the up-regulated list, the activation of genes for proteins of the JAK-STAT signalling pathway, which may result in increased expression of Mx proteins, and of Vig-2 and cholesterol 25-hydroxylase, the latter being an enzyme of steroid biosynthesis activated with type I IFNs in mammalian macrophages [46], indicate molecular mechanisms inducing immune pathways in ASRV infection. Consequences of activation of this pathway could include stimulation of natural killer cells (NK), confirmed in our list by strong up-regulation of granzyme A, and receptors found in NK cells such as CD229, and T cell receptor α. Activation of IFN I could lead to increased expression of major histocompatibility complex (MHC) class I to promote antigen presentation activation. However, although genes related to MHC I such as beta-2 microglobulin and TAP proteins were up-regulated (see Table 6), it appears that activation of this pathway may not be as important in ASRV infection as it is in other viral infections such as ISAV [47]. The previous ISAV study was performed at early stages of the infection in comparison to the much longer time scale of the present study, and so this may indicate that the importance or role of different pathways varies during the progression of the infection, as would be expected. The activation of NK cells, the prominent up-regulation of genes associated with cytotoxic T cells (T-cell receptor α and β chains, CD97 and CD9), and up-regulation of interleukins 12 and 18 will likely result in increased IFN II (IFN-γ), which was also shown to be up-regulated. This up-regulation is in agreement with the induction of the IFN II responses reported previously in viral infections of salmon, specifically ISAV [47], IPNV [48] and PMCV [44]. As was reported previously, the activation of the cytotoxic T cells after infection with PMCV seems to be a prominent feature of the immune system for clearance of the viral infection [44]. As LC-PUFA have been shown to modulate the production of IFN-γ, [49], the differences in the levels of EPA and in the EPA/ARA ratio between the diets may contribute to the differential immune responses observed although, as described above, the differences in LC-PUFA between the two functional feeds were relatively small.

The present study also showed up-regulation of MHC class II that may indicate an important humoral immune response to ASRV. In contrast, up-regulation of this gene was not observed in salmon in response ISAV at early stage infection [47]. The 70-fold higher expression of MHC class II in fish fed the FF1 diet at 16-weeks post-infection, when viral load was high, compared with those fed the ST diet, could be indicative of a more efficient response against ASRV at that time in fish fed FF1.

In general, the expression of all genes characteristic of the immune response was different between the dietary treatments, not only in terms of the time-courses of ASRV infection and response, but also when comparing gene expression in fish presenting high viral load, with consistently lower expression in fish fed the FF1 diet. The less extreme effects on immune gene expression, especially those of the innate immune response, may be indicative of a milder/controlled response in fish fed the FF1 diet, and the reason why fish on this treatment presented fewer and less severe heart lesions. This is consistent with previous data on salmon challenged with ISAV that showed fish with the highest expression of genes involved in the innate immune response were those with low resistance to the disease [25]. Therefore, it appears that in viral diseases like HSMI, with lower mortality but high morbidity, the control of over-expression of genes involved in antiviral host responses could be key to better performance of the fish.

Leukotriene LTB_4_ plays important roles in the immune response, enhancing NK cell activity, stimulating lymphocyte production of IFN-γ, and modulating immune cell function promoting the proliferation of T cells and stimulating the release of cytokines from those cells [21], [50]. As LTB_4_ is synthesized from ARA derived from cell membrane phospholipids, it represents a link between tissue LC-PUFA profiles and the expression of the immune related genes mentioned above. Two genes in the up-regulated list related to LTB_4_ metabolism, arachidonate 5-lipoxygenase (LTB_4_ biosynthesis) and leukotriene B_4_ receptor, were up-regulated two-fold when the viral load was high in fish fed the ST and FF2 diets compared with fish fed the FF1 diet. This is one example of how, early in the cascade of events of the immune response, differences can be found between fish fed the FF1 diet and fish fed the other diets. Differential expression of both these genes was also reported after infection with ISAV [25], thus the eicosanoid pathways appear to be important in salmon viral diseases, determining the magnitude of the inflammatory response.

The present study has clearly demonstrated that application of clinical nutrition and the use of functional feeds can have a major influence on the development and severity of the response to ASRV infection in salmon. Furthermore, the differences in gene expression between fish fed the different diets have provided further insight into the molecular mechanisms and progression of the immune responses to ASRV infection in salmon. However, the precise mechanisms underpinning both the delayed replication of the virus in fish fed the functional feeds and the differential effects of these two formulations require further investigation.

## Conclusion

The present study is the first to describe the effects of functional feeds on the expression of genes related to the immune response after infection with ASRV. The long duration of the experiment allowed us to evaluate the host-pathogen interaction, increasing knowledge of HSMI, an important emerging disease in Atlantic salmon aquaculture. Clear differences on viral load and the immune response were found in fish fed the different dietary treatments, highlighting the immune modulatory role of dietary lipid content and composition in viral infections. Reduction in dietary lipid along with increased EPA can lead to a milder inflammatory response and consequently less severity of the heart lesions caused by ASRV infection indicating that dietary immunomodulation could reduce the morbidity of the disease improving the performance of fish suffering HSMI.

## Materials and Methods

### Fish and experimental feeds

Three fishmeal-based diets were formulated and manufactured by EWOS Innovation (Dirdal, Norway) (Table 12). The reference diet (ST) was essentially a standard, commercial formulation with 31% lipid with the added oil being a blend of FO and rapeseed oil. The two functional feeds (FF) both contained a lower level of lipid that was balanced by increased protein, provided by fishmeal and krill meal. Both also contained increased proportions of EPA, but with different levels of n-6 PUFA and DHA, resulting in the n-3/n-6 PUFA ratio in the feeds varying from 1.5 to 3.6, through the inclusion of krill oil in one of the feeds replacing rapeseed oil (Table 3). The precise formulations were based on small-scale commercial screening trials (unpublished). Therefore, as one major factor being investigated was dietary lipid level, the diets were not isolipidic or isoproteic. The diets were termed ST (standard reference diet), FF1 and FF2.

**Table 12:** Formulation of the experimental diets.

Component %^1^	STD	FF1	FF2
Fish meal and hydrolysates	42.1	53	53
Fish oil	13.7	7	5.2
Vegetable protein concentrates^2^	21	18	18
Vegetable oil	11.2	3	0
Carbohydrate-based binders^3^	11.2	12.1	12.1
Micro premixes^4^	0.8	1.9	1.7
Krill meal^5^	0	5	5
Krill oil^5^	0	0	5
Total	100	100	100
**Proximate composition**			
Moisture	6.5	6.5	6.5
Fat	31	18	18
Protein	42.2	53.4	53.4

1) All ingredients sourced from EWOS stocks unless otherwise stated.

2) Includes soy protein concentrate, pea protein concentrate, wheat gluten and sunflower meal.

3) Includes wheat grain.

4) Includes vitamins, minerals, crystalline amino acids, ammonium phosphate.

5) Aker Biomarine A.

A total of 450 Atlantic salmon (*Salmo salar* L.), unvaccinated AquaGen strain, were distributed into three tanks at the EWOS facility, Lønningdal, Norway and fed one of the three feeds for a period of 8 weeks. After this pre-challenge feeding phase, 390 (130 fish per treatment) fish (non-vaccinated) with an initial average weight 220 g (±3.2 g, standard error), were transferred to the challenge facility at the Industrial and Aquatic Laboratory (ILAB), Bergen, Norway. Fish were distributed into two independent experimental rooms supplied with filtered sea water (of approximately 30‰) each containing 9 tanks (3 tanks per dietary treatment in each system). Water delivery was flow-through, with a supply to maintain oxygen concentration in outlet water >8 mg/L. Water temperature was maintained at 10±1°C, water flow rate was 0.8 L/kg fish/min, and a 12∶12 h light/dark regime was followed. The fish were acclimated for 2 weeks prior to challenge and were fed with the same diets during the acclimation period and throughout the period of the challenge (16 weeks) that they were fed prior to transfer. No previous diseases were described. Ten fish from each feeding group (n = 30 in total) were sampled prior to challenge (0 time-point controls) for histological examination to confirm they were disease-free.

### ASRV Challenge

As this was a nutritional trial designed to test the effects of different feeds, all 360 remaining fish were challenged with pathogen. The fish were sedated using Aqui-S at final concentration of 5 mg/L of isoeugenol, followed by anaesthesia in Benzoak using a final concentration of 30 ml/L of water. Fish were challenged by intramuscular injection (0.1 ml on each side close to the lateral line) of ASRV collected from cell culture supernatant of piscine reovirus (see below). There were no mortalities during the trial, which was consistent with previous experimental studies of HSMI [1], [5].

### Virus isolation and preparation of inoculum

Heart tissue, collected from a clinical outbreak of HSMI, was homogenized and centrifuged 3500×g for 20 min 4°C to remove cellular debris and then filtered (0.45 µm). The homogenate was inoculated onto GF-1 cells derived from fin tissue of orange-spotted grouper, *Epinephelus coioides*. This was done in collaboration with PHARMAQ AS, Oslo, Norway who supplied the cell line. The cells were grown at 15°C in Leibovitz L-15 medium (Invitrogen AS, Oslo, Norway) supplemented with 1% L-glutamine, 0.1% gentamicin sulphate (Sigma Aldrich Norway AS, Oslo, Norway) and 10% fetal bovine serum (Invitrogen). At 2 weeks after inoculation the supernatant and cell lysate were harvested and cleared by centrifugation at 3500×g for 20 min at 4°C.

### Sampling

Ten fish, chosen at random, from each dietary treatment group (5 fish from each of the two independent systems) were sampled at 8-, 10-, 12-, and 16-weeks post-challenge for histology, lipid and biomolecular analysis (data presented). However, in addition a further 10 fish per treatment were sampled at 14 weeks post-challenge for histology (data not shown) and all remaining fish were culled at week 16 post-challenge for possible further histological analysis. Fish were anaesthetized and killed by a blow to the head, and heart and head kidney collected for analyses. Part of each heart sample was transferred to 10% buffered formalin for histological analyses and another part was immediately frozen in liquid N_2_ and stored at −80°C prior to lipid and molecular analyses. Head kidney was immediately frozen in liquid N_2_ and stored at −80°C.

### Histological examinations

Inflammatory response in ASRV challenged fish was assessed by histological changes in heart tissue (epicardium and myocardium) as assessed by light microsopy. The scoring system marked samples on a visual analog scale on the basis of the criteria given in Table S3 [3]. A maximum score of 6 can be achieved for combined epicardial and myocardial changes. All evaluations were carried out blind. Further, details of the different feeds were not revealed to the histological examiner (ØE), i.e. the histological examination was carried out as a double-blind study.

Histological changes in epicardium and myocardium were ranked according to a non-continuous score grade from 0 to 4 (0 indicates no pathology, normal tissue; 4 intense inflammation). A description of each of the scores is included in Table S3.

All data preparation and simulation output analysis was conducted with the R language [51]. The model was a mixed-effects linear model estimated with the lmer function in the lme4 package. The treatment estimates were based on posterior simulation (n = 2500) with 95% credible intervals as absolute and proportional to the reference level (control diet).

Histopathological scores were analysed by using a multilevel ordered categorical logistic regression because the data are multinomial. The model was written in BUGS [52] language and fitted with JAGS [53]. Vague non-informative uniform priors [0,100] were given for the variance parameters and vague non-informative normal priors N (0, 1.0E+4) for all other parameters. 25000 “burn-in” simulation runs were used to adapt the Markov Chain Monte Carlo (MCMC) before subsequent 2500 runs that were used for inference. Three chains were run in parallel, i.e. there were a total of 7500 simulations for inference. These were thinned so that only every 10th simulation was saved to reduce the size of saved objects and to reduce the effects of autocorrelation. In effect, the posterior density is based on 750 draws from the posterior probability distribution. Convergence of the MCMC simulation was judged by the so-called Gelman-Rubin convergence diagnostic [54].

### Lipid analyses

Lipid and fatty acid analyses were performed on heart and head kidney tissue samples from five fish per treatment at 8- and 16-weeks post-challenge. Total lipid from approximately 1 g of heart and head kidney tissue was extracted by homogenization in chloroform/methanol (2∶1, by volume) according to Folch et al. [55], and determined gravimetrically. Fatty acid methyl esters (FAME) of total lipid were prepared by acid-catalyzed transmethylation [56], and FAME separated and quantified by gas chromatography as described in detail previously [40]. Tissue and diet lipid class compositions were determined by single-dimension double-development high-performance thin-layer chromatography (HPTLC) and densitometry as described previously [40]. Significance of differences due to diet and time were determined by two-way ANOVA (p<0.05) using the SPSS 19.0 statistical package (SPSS Inc., Chicago IL, USA). Significance of differences in heart fatty acid compositions due to diet at each time point were determined by one-way ANOVA (p<0.05) (SPSS).

### RNA extraction and purification

Heart tissue from six individuals per dietary treatment was rapidly homogenized in TRI Reagent (Ambion, Applied Biosystems, Warrington, U.K.) using an Ultra-Turrax homogenizer (Fisher Scientific, Loughborough, U.K.). Total RNA was isolated following manufacturer's instructions, and RNA quality and quantity assessed by gel electrophoresis and spectrophotometry (NanoDrop ND-1000, Thermo Scientific, Wilmington, U.S.A.), respectively. One hundred micrograms of total RNA from each individual sample was further purified by mini spin-column purification (RNeasy Mini Kit, Qiagen, Crawly, UK), and quantified and quality assessed as above.

### Microarray hybridizations and analysis

The transcriptomic experiment used an Atlantic salmon custom-made oligoarray with 44 k features per array on a four-array-per-slide format, with each feature printed singly (Agilent Technologies UK Ltd., Wokingham, UK). The probes were co-designed by researchers at the Institute of Aquaculture, University of Stirling, U.K. and The Norwegian Institute of Food, Fisheries and Aquaculture Research (Nofima, Tromsø, Norway), and array design is available on request. A dual-labelled experimental design was employed for the microarray hybridizations. Each experimental sample was competitively hybridized against a common pooled-reference sample, which comprised equal amounts of each of the replicates used in the study. This design permits valid statistical comparisons across all treatments to be made. The entire experiment comprised 72 hybridizations; 4 time points (8, 10, 12 and 16 weeks)×3 diets (ST, FF1 and FF2)×6 biological replicates.

Indirect labelling methodology was employed in preparing the microarray targets. Amplified antisense RNA (aRNA) was produced from each RNA sample using the Amino Allyl MessageAmpTM II aRNA Amplification Kit (Ambion, Applied Biosystems, Warrington, UK), following the manufacturer's methodology, followed by Cy3 or Cy5 fluor incorporation through a dye-coupling reaction. Briefly, 500 ng of total RNA per sample were amplified and column-purified according to manufacturer's instructions including a 17 h transcription step. Resultant aRNA was quantified and quality assessed as above. Subsequently, Cy dye suspensions (Cy3 & Cy5) in sufficient quantity for all labelling reactions were prepared by adding 36 µL high purity dimethyl sulphoxide (Stratagene, Agilent Technologies UK Ltd.) to each tube of Cy dye (PA23001 or PA25001, GE HealthCare, Chalfont St. Giles, UK). To attach the Cy dyes, 3 µg each aRNA sample was suspended in 10 µL nuclease-free H_2_O and heated to 70°C for 2 min. When cooled to room temperature, 3 µL of coupling buffer (0.5 M NaHCO_3_; pH 9.2) and 2 µL of Cy3 dye suspension stock was added and then incubated for 1 h at 25°C in the dark. To label the common pooled reference sample with Cy5, a scaled-up batch reaction was similarly performed. Unincorporated dye was removed by column purification (Illustra AutoSeq G-50 spin columns; GE Healthcare). Dye incorporation and aRNA yield were quantified by spectrophotometry (NanoDrop) and further quality controlled by separating 0.4 µL of the sample on a thin mini-agarose gel and visualising products on a fluorescence scanner (Typhoon Trio, GE Healthcare).

Hybridization of a total of 6 slides (24 arrays) was performed in a single day, with sample order semi-randomized, using SureHyb hybridization chambers in a DNA Microarray Hybridization Oven (Agilent Technologies). For each hybridization, 825 ng of Cy3-labelled experimental biological replicate and Cy5-labelled reference pool were combined and total volume made up to 38 µl with nuclease-free water. A fragmentation master mix was prepared containing, per reaction, 11 µl 10× blocking agent, 2.2 µl 25× fragmentation buffer and 6.8 µl nuclease-free water, and 20 µl was dispensed into the Cy-dyes mix. After incubating in the dark at 60°C for 30 min, 57 µl 2×GE Hybridization buffer (pre-heated to 37°C) was added, contents gently mixed, spun at 16,000 g for 1 min and kept on ice until loaded onto the microarray slides as per the manufacturers protocol. Hybridization was carried out in the oven rotator (Agilent Technologies) at 65°C and 10 rpm for 17 h. Post-hybridization washes were carried out in EasyDip™ Slide staining containers (Canemco Inc., Quebec, Canada). After disassembling the array-gasket sandwiches submersed in wash buffer 1 at room temperature, the microarray slides were transferred to an EasyDip™ container and incubated in wash buffer 1 for 1 min at 31°C in an orbital incubator rotating at 150 rpm, and then a further 1 min at 31°C at 150 rpm in wash buffer 2. A final dip in wash buffer 2 at room temperature was performed, after which the slides were dried by centrifugation (500×g for 6 min) and kept in a desiccator in the dark until scanned the same day. Unless otherwise stated, all reagents were from Agilent Technologies.

Scanning was performed at 5 µm resolution using an Axon GenePix 4200AL Scanner (MDS Analytical Technologies, Wokingham, Berkshire, U.K.). Laser power was kept constant (80%) and the “auto PMT” function within the acquisition software (v.4) was enabled to adjust PMT for each channel such that less than 0.1% of features were saturated and that the mean intensity ratio of the Cy3 and Cy5 signals was close to one. Agilent Feature Extraction Software (v 9.5) was used to identify features and extract fluorescence intensity values from the resultant TIF images. The remaining analysis was then performed in the GeneSpring GX version 10.0.2 analysis platform (Agilent Technologies). All intensity values <0.1 were set to 0.1 followed by a block Lowess normalisation. After removing control features, a series of four quality filtering steps was carried out sequentially using a range of quality control metrics produced by the Agilent Feature Extraction software to remove features that were saturated, non-uniform, population out layers and spots non-significantly different from background.

Hybridization data were analyzed by two-way ANOVA, which examined the explanatory power of the variables ‘diet’ and ‘time’ and the interaction between the two, at a significance level of 0.05. In the present study we focussed on immune-related genes whose expression was differentially affected by diet at 16 weeks post-challenge and thus only data from the significant interaction list was analyzed. That list contained 2584 genes, including repeated probes. Due to the large size of the list, hierarchical cluster analysis and 5×5 Self-Organizing Maps cluster analysis (SOM) were used to map the common temporal expression profiles of the genes from the list using the GeneSpring GX version 10.0.2 analysis platform (Agilent Technologies).

### RT-qPCR

Expression of 12 selected genes showing a significant diet×time interaction in the microarray analysis and related to relevant immune-related pathways was studied by reverse transcription quantitative real-time PCR (qPCR). The qPCR primer sequences, annealing temperature (Tm) and size of amplicon are given in Table S4. The sequences were obtained either by literature searches or designed from EST sequences corresponding to microarray clones or candidate genes of interest using Primer3 software (http://biotools.umassmed.edu/bioapps/primer3_www.cgi). In addition, amplification of three potential reference genes, *cofilin-2*, elongation factor-1α (*elf-1α*) and *β-actin*, was performed but only *cofilin-2* and *elf-1α* expression was sufficiently stable across treatments for normalization. *Cofilin-2* and *elf-1α* had been identified in previous salmon cDNA microarray and qPCR studies as suitable reference genes on the basis of constant expression between different feeds and time points [57], [58].

For qPCR, 2 µg of column-purified total RNA per sample was reverse transcribed into cDNA using the High-Capacity cDNA RT kit (Applied Biosystems, Paisley, U.K.), following manufacturer's instructions, but using a mixture of the random primers (1.5 µl as supplied) and anchored oligo-dT (0.5 µl at 400 ng/µl, Eurofins MWG Operon, Ebersberg, Germany). Negative controls (containing no enzyme) were performed to check for genomic DNA contamination. cDNA was then diluted 20-fold with water, after a similar amount of cDNA was pooled from all samples. qPCR analysis used relative quantification with the amplification efficiency of the primer pairs being assessed by serial dilutions of the cDNA pool. qPCR amplifications were carried out in duplicate (Quantica, Techne, Cambridge, U.K.) in a final volume of 20 µL containing either 5 µL (for most genes) or 2 µL (for the reference genes and other highly expressed genes) diluted (1/20) cDNA, 0.5 µM of each primer and 10 µL AbsoluteTM QPCR SYBR® Green mix (ABgene). Amplifications were carried out with a systematic negative control (NTC-non template control, containing no cDNA). The qPCR profiles contained an initial activation step at 95°C for 15 min, followed by 30 to 40 cycles: 15 s at 95°C, 15 s at the specific primer pair annealing Tm and 15 s at 72°C. After the amplification phase, a melt curve of 0.5°C increments from 75°C to 90°C was performed, enabling confirmation of amplification of single products, and sizes were checked by agarose gel electrophoresis and identities confirmed by sequencing. Non-occurrence of primer-dimer formation in the NTC was also verified. Data were analyzed using the relative expression software tool [REST 2009, http://www.gene-quantification.info/], which employs a pair wise fixed reallocation randomization test (10,000 randomizations) with efficiency correction, to determine the statistical significance of expression ratios (or gene expression fold-changes) between two treatments [59].

### Assessment of viral load

Relative quantification of ASRV by qPCR was used to determine differences in the viral load between the different dietary treatments at the two critical points in the time-course of the infection (12- and 16- weeks post-challenge) when differential expression of genes related to the inflammatory process was greatest between fish fed the ST diet and fish fed the functional feeds. The qPCR primer sequences (Table S4) previously described by Mikalsen et al. [3] were employed following the same procedure and from the same RNA samples used for the validation of the microarray (as described above).

## Supporting Information

Figure S1 Normalized gene expression levels in heart for different gene groups. The genes listed in Tables 4–9 are included in figures A–F, showing the average gene expression levels (as whiskers), with maximum and minimum range for each gene group. Outliers are depicted as black dots. A) Viral infection-related genes; B) Innate immune system-related genes; C) IFN I related-genes; D) Antiviral host responses related-genes; E) IFN II-related genes; and F) Adaptive immune system related-genes. P values are depicted (One-way Anova) and n is the same as in corresponding Tables 4–9.
(TIF)

Table S1 Complete list of the down-regulated genes at 12wpc on the group of fish fed with the ST diet and 16wpc on the group of fish fed with the FF2 diet from cluster groups (identified by SOM).
(XLSX)

Table S2 Complete list of the down-regulated genes at 12wpc on the group of fish fed with the ST diet and 16wpc on the group of fish fed with the FF2 diet from cluster groups (identified by SOM).
(XLSX)

Table S3 Criteria used to score the histological changes in the heart (epicard, ventricle and atrium).
(DOCX)

Table S4 Primers used for RT-qPCR analyses.
(DOCX)
