# Supplementary figures and images for: Functional Feeds Reduce Heart Inflammation and Pathology in Atlantic Salmon (Salmo salar L.) following Experimental Challenge with Atlantic Salmon Reovirus (ASRV)
Source: PLoS One. 2012 Nov 30;7(11):e40266. doi: 10.1371/journal.pone.0040266 (PMC3511526; doi:10.1371/journal.pone.0040266)

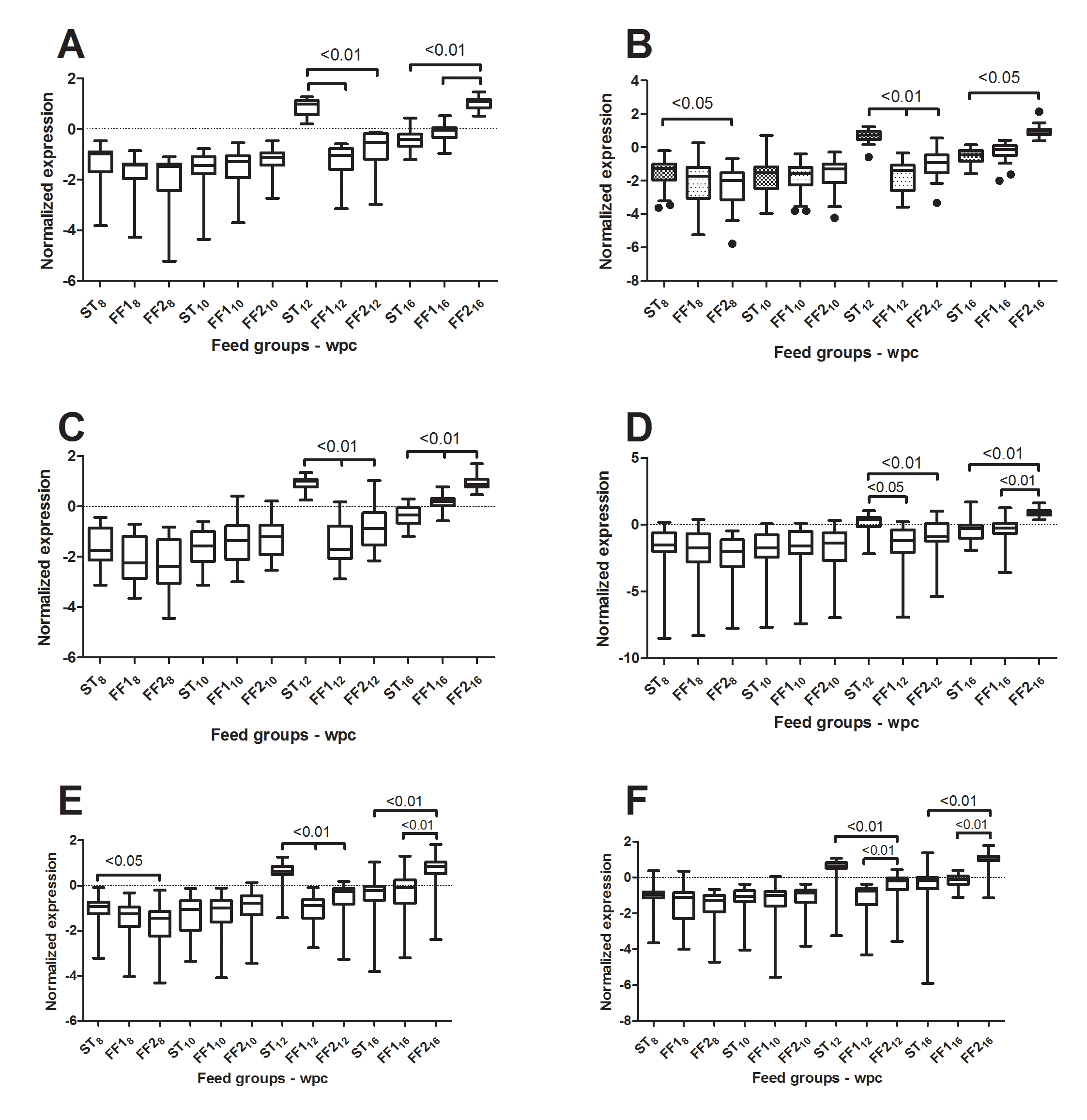

Supplement: Figure S1 — Normalized gene expression levels in heart for different gene groups. The genes listed in Tables 4–9 are included in figures A–F, showing the average gene expression levels (as whiskers), with maximum and minimum range for each gene group. Outliers are depicted as black dots. A) Viral infection-related genes; B) Innate immune system-related genes; C) IFN I related-genes; D) Antiviral host responses related-genes; E) IFN II-related genes; and F) Adaptive immune system related-genes. P values are depicted (One-way Anova) and n is the same as in corresponding Tables 4–9. (TIF) [file pone.0040266.s001.tif]
