# Supplementary material for: Functional Feeds Reduce Heart Inflammation and Pathology in Atlantic Salmon (Salmo salar L.) following Experimental Challenge with Atlantic Salmon Reovirus (ASRV)
Source: PLoS One. 2012 Nov 30;7(11):e40266. doi: 10.1371/journal.pone.0040266 (PMC3511526; doi:10.1371/journal.pone.0040266)
Supplement: Table S3 — Criteria used to score the histological changes in the heart (epicard, ventricle and atrium). (DOCX) [file pone.0040266.s004.docx]

**Table S3**. Criteria used to score the histological changes in the heart (epicard, ventricle and atrium).

| **Inflammation score for HSMI infected fish** | **Pathological description - epicard** | **Pathological description - myocard** |
| --- | --- | --- |
| 0 | No pathological changes observed. | No pathological changes observed. |
| 0-1.8 | **Score 0.1-0.9**: Focal / multifocal (2-4 foci) of inflammatory cells lifting the epicardial layer from the surface of the heart, typically 2-3 cell layers thick. Limited number (countable) of mononuclear inflammatory cells infiltrating the epicardium.  If there is only involvement of epicard with minor or very little compact layer involvement; max 1.5 score (diffuse and >5 cell layer thick for most of the inflamed area). | **Score 0.1-0.9**. Vascular changes in the small vessels of the compact layer characterized by enlarged endothelial cells, typically stretching out. Minor inflammatory changes of the compact layer without significant involvement of the spongious layer. |
| 2-3.8 | **Score 1-1.9**: Diffuse infiltration of inflammatory cells (mononuclear) >5 cell layers thick in most of the epicard present. The infiltration of cells is multifocal to diffuse and can involve parts of or the entire epicardium available for assessment. | **Score 1-1.9:** Focal to multifocal inflammatory foci (2-5 foci) of the compact layer and/or in the spongious part (2-5 foci). Extension typically seen along small vessels and perivascular infiltration. |
| 4-5.8 | **Score 2.0-2.9**: Diffuse infiltration of inflammatory cells (mononuclear) >10 cell layers thick in most of the epicard present. Moderate pathological changes consisting of high number (uncountable) of inflammatory cells in the epicardium | **Score 2.0-2.9:** The changes in the compact layer are multifocal or diffuse in areas and typically concentrate along small blood vessels. Combined with focal or multifocal changes in the spongious layer. |
| 6 | **Score 3**: Diffusely thickened (>15 cell layers) epicard in more than ¾ of the layer present. Severe pathological changes characterized by intense infiltration of inflammatory cells in the epicardium,. | **Score 3:** Widespread to diffuse infiltration of inflammatory cells in the compact layer and involving the spongius layer in a multifocal pattern. Degeneration and or necrosis of muscle fibers may be/are seen. Atrium can also be involved with inflammatory changes |

Max score within each category is given in the left-most column. Scoring was done on a visual analogue scale.
