# Supplementary material for: Functional Feeds Reduce Heart Inflammation and Pathology in Atlantic Salmon (Salmo salar L.) following Experimental Challenge with Atlantic Salmon Reovirus (ASRV)
Source: PLoS One. 2012 Nov 30;7(11):e40266. doi: 10.1371/journal.pone.0040266 (PMC3511526; doi:10.1371/journal.pone.0040266)
Supplement: Table S4 — Primers used for RT-qPCR analyses. (DOCX) [file pone.0040266.s005.docx]

| Transcript | Primer name | Primer sequence | Fragment | Tm | Accession No. | Source |
| --- | --- | --- | --- | --- | --- | --- |
| *IL10* | IL10-F1 | 5’- AAGGCAGTGTTCGAGAGCAT -3’ | 162 bp | 60º | BT047745 ^1^ | New design |
|  | IL10-R1 | 5’- CTTCCCCATCCACTTCAAGA -3’ |  |  |  |  |
| *Casp14* | CASP14-F1 | 5’- TGGAGAGATCACAGGAGAGGA -3’ | 166 bp | 60º | BT048360 ^1^ | New design |
|  | CASP14-R1 | 5’- GGAAGCAAAATGGAACGAAA -3’ |  |  |  |  |
| *TCRa* | TCRa-F1 | 5’- GCCTGGCTACAGATTTCAGC -3’ | 220 bp | 60ºC | BT050114 ^1^ | New design |
|  | TCRa-R1 | 5’- CCAGAATGGTCAGGGATAGG -3’ |  |  |  |  |
| *IRF1* | IRF1-F2 | 5’- CGGTCACCAAGAAACCCTTA -3’ | 386 bp | 60ºC | EF067841 ^1^ | New design |
|  | IRF1-R2 | 5’- CGCAGCTCTATTTCCGTTTC -3’ |  |  |  |  |
| *MX1* | MX1-F2 | 5’- CTGCAGAACAAGCTCAAACG -3’ | 171 bp | 60ºC | U66475 ^2^ | New design |
|  | MX1-R2 | 5’- TCCTCTGGGTCCACATTGTA -3’ |  |  |  |  |
| *INFII* | INFII-F2 | 5’- TTCAGGAGACCCAGAAACACTAC -3’ | 125 bp | 60ºC | AJ841811 ^1^ | S.M. Jørgensen et al. 2007 |
|  | INFII-R2 | 5’- TAATGAACTCGGACAGAGCCTTC-3’ |  |  |  |  |
| *GIG2* | GIG2-F | 5’- CCCCTGAGGACAGCCACGTCT -3’ | 114 bp | 60ºC | EG815123^1^ | New design |
|  | GIG2-R | 5’- CACCGGCACCAAGCATGCCA -3’ |  |  |  |  |
| *SAA* | SAA-F | 5’- GGGAGATGATTCAGGGTTCCA -3’ | 79 bp | 60ºC | X99387 ^2^ | M.K. Raida, K. Buchmann 2009 |
|  | SAA-R | 5’- TTACGTCCCCAGTGGTTAGC -3’ |  |  |  |  |
| FLAP | FLAP-F | 5’- TCTGAGTCATGCTGTCCGTAGTGGT -3’ | 111 bp | 60ºC | CA369467 ^1^ | Jørgensen et al. (2008) |
|  | FLAP-R | 5’- CCTCCCTCTCTACCTTCGTTGCAAA -3’ |  |  |  |  |
| BAF | BAF-F2 | 5’- GCAGACAGTCACCTTCTCTCC -3’ | 187 bp | 60ºC | BT049316 ^1^ | New design |
|  | BAF-R2 | 5’- GGGTACAAGCAGGGGTCTTA -3’ |  |  |  |  |
| B2M | B2M-F | 5’- TCCCAGACGCCAAGCAG -3’ | 138 bp | 55ºC | AF180487 ^1^ | N.D. Young et al. (2008) |
|  | B2M-R | 5’- TGTAGGTCTTCAGATTCTTCAGG -3’ |  |  |  |  |
| *IgER* | IgER-F1 | 5’- GGGAAGTTGAGCTGTGGGTA -3’ | 201 bp | 60ºC | BT048133 ^1^ | New design |
|  | IgER-R1 | 5’- AGCGCCATAGAAGCTTTGAA -3’ |  |  |  |  |
| *ASRV* | ASRV-F1 | 5’- CGTACCGCTTCTAACCAAGC -3’ | 215 bp | 58ºC | HM453201 ^1^ | Mikalsen et al.(2012) |
|  | ASRV-R1 | 5’- ACATGACGACGGACTCCAAT -3’ |  |  |  |  |
| *Reference genes:* | |  |  |  |  |  |
| *elf-1α* | ELF-1A jbt2 | 5’-CTGCCCCTCCAGGACGTTTACAA-3’ | 175 bp | 60ºC | AF321836 ^1^ | Morais et al. (2011) |
|  | ELF-1A jbt2 | 5’-CACCGGGCATAGCCGATTCC-3’ |  |  |  |  |
| *β-actin* | BACT-F | 5’-ACATCAAGGAGAAGCTGTGC-3’ | 141 bp | 56ºC | AF012125 ^1^ | Morais et al. (2011) |
|  | BACT-R | 5’-GACAACGGAACCTCTCGTTA-3’ |  |  |  |  |
| *Cofilin-2* | B2F | 5’-AGCCTATGACCAACCCACTG-3’ | 224 bp | 60ºC | TC63899 ^2^ | Morais et al. (2011) |
|  | B2R | 5’-TGTTCACAGCTCGTTTACCG-3’ |  |  |  |  |

^1^ GenBank (<http://www.ncbi.nlm.nih.gov/>)

^2^ Atlantic salmon Gene Index (<http://compbio.dfci.harvard.edu/tgi/>)

References used in this table:

Jørgensen SM, Hetland DL, Press CM, Grimholt U, Gjøen T (2007) Effect of early infectious salmon anaemia virus (ISAV) infection on expression of MHC pathway genes and type I and II interferon in Atlantic salmon (Salmo salar L.) tissues. Fish Shellfish Immunol 23: 576-588.

Jørgensen S, Afanasyev S, Krasnov A (2008) Gene expression analyses in Atlantic salmon challenged with infectious salmon anemia virus reveal differences between individuals with early, intermediate and late mortality. BMC Genomics 9: 179.

Mikalsen AB, Haugland O, Rode M, Solbakk IT, Evensen Ø (2012) Atlantic salmon reovirus infection causes a CD8 T cell myocarditis in Atlantic salmon (Salmo salar L.). PLOS One, in press.

Morais S, Pratoomyot J, Taggart JB, Bron JE, Guy DR, Bell JG, Tocher DR (2011) Genotype-specific responses in Atlantic salmon (Salmo salar) subject to dietary fish oil replacement by vegetable oil: A liver transcriptomic analysis. BMC Genomics 12: 255.

Raida, M. K., & Buchmann, K. (2009). Innate immune response in rainbow trout (oncorhynchus mykiss) against primary and secondary infections with yersinia ruckeri O1. Developmental and Comparative Immunology, 33(1), 35-45.

Young, N. D., Cooper, G. A., Nowak, B. F., Koop, B. F., & Morrison, R. N. (2008). Coordinated down-regulation of the antigen processing machinery in the gills of amoebic gill disease-affected atlantic salmon (salmo salar L.). Molecular Immunology, 45(9), 2581-2597.
